# Different developmental histories of beta-cells generate functional and proliferative heterogeneity during islet growth

**DOI:** 10.1038/s41467-017-00461-3

**Published:** 2017-09-22

**Authors:** Sumeet Pal Singh, Sharan Janjuha, Theresa Hartmann, Özge Kayisoglu, Judith Konantz, Sarah Birke, Priyanka Murawala, Ezzaldin Ahmed Alfar, Kei Murata, Anne Eugster, Naoki Tsuji, Edward R. Morrissey, Michael Brand, Nikolay Ninov

**Affiliations:** 1 DFG-Center for Regenerative Therapies Dresden, Cluster of Excellence, Technische Universität Dresden, Dresden, 01307 Germany; 2 Paul Langerhans Institute Dresden of the Helmholtz Zentrum München at the University Hospital and Faculty of Medicine Carl Gustav Carus of Technische Universität Dresden, Dresden, 01307 Germany; 3 Pain & Neuroscience Lab, Daiichi Sankyo Co., Ltd., Tokyo, 140-8710 Japan; 4 Weatherall Institute of Molecular Medicine, Oxford, OX3 9DS UK; 5 German Center for Diabetes Research (DZD e.V.), Neuherberg, Neuherberg, 85764 Germany

## Abstract

The proliferative and functional heterogeneity among seemingly uniform cells is a universal phenomenon. Identifying the underlying factors requires single-cell analysis of function and proliferation. Here we show that the pancreatic beta-cells in zebrafish exhibit different growth-promoting and functional properties, which in part reflect differences in the time elapsed since birth of the cells. Calcium imaging shows that the beta-cells in the embryonic islet become functional during early zebrafish development. At later stages, younger beta-cells join the islet following differentiation from post-embryonic progenitors. Notably, the older and younger beta-cells occupy different regions within the islet, which generates topological asymmetries in glucose responsiveness and proliferation. Specifically, the older beta-cells exhibit robust glucose responsiveness, whereas younger beta-cells are more proliferative but less functional. As the islet approaches its mature state, heterogeneity diminishes and beta-cells synchronize function and proliferation. Our work illustrates a dynamic model of heterogeneity based on evolving proliferative and functional beta-cell states.

## Abstract (web summary)

Βeta-cells have recently been shown to be heterogeneous with regard to morphology and function. Here, the authors show that β-cells in zebrafish switch from proliferative to functional states with increasing time since β-cell birth, leading to functional and proliferative heterogeneity.

## Introduction

Organ growth, with its goal for increasing tissue size while sustaining physiological demands, is driven by the differentiation of stem cells, as well as by the replication of pre-existing differentiated cells^1, 2^. Organs such as the brain or the intestine rely on the tissue-resident stem cells to increase the pool of differentiated cells that perform the organ’s function. In contrast, the heart, liver, and pancreas increase their cellular mass in part by the replication of differentiated cells that also perform the organ’s function^3–5^. It remains unknown if the contributions to growth and function are equally shared among differentiated cells, or if these properties are allocated to different populations. In an “egalitarian” strategy, all cells could contribute equally to both growth and function. Alternatively, cells could divide these two tasks to heterogeneous populations with different proliferative and functional capacities.

Organized within micro-organs called the islets of Langerhans, the pancreatic beta-cells provide an intriguing model for studying the allocation of proliferative and functional tasks. Since insulin plays an indispensable role in maintaining blood glucose levels, continuous insulin production and secretion need to be delicately balanced with the energetically demanding task of cell division^6^, which is important for increasing the beta-cell mass^4^. Recently, the heterogeneity among beta-cells has become strikingly evident^7–14^, as elegant studies identified sub-populations of beta-cells based on topological location^15^, cell-surface markers,^16, 17^ or gene-expression^18–20^. However, the factors contributing to the diversity among beta-cells remain to be identified. In particular, the developmental source of heterogeneity remains an open question.

**Fig. 1:**
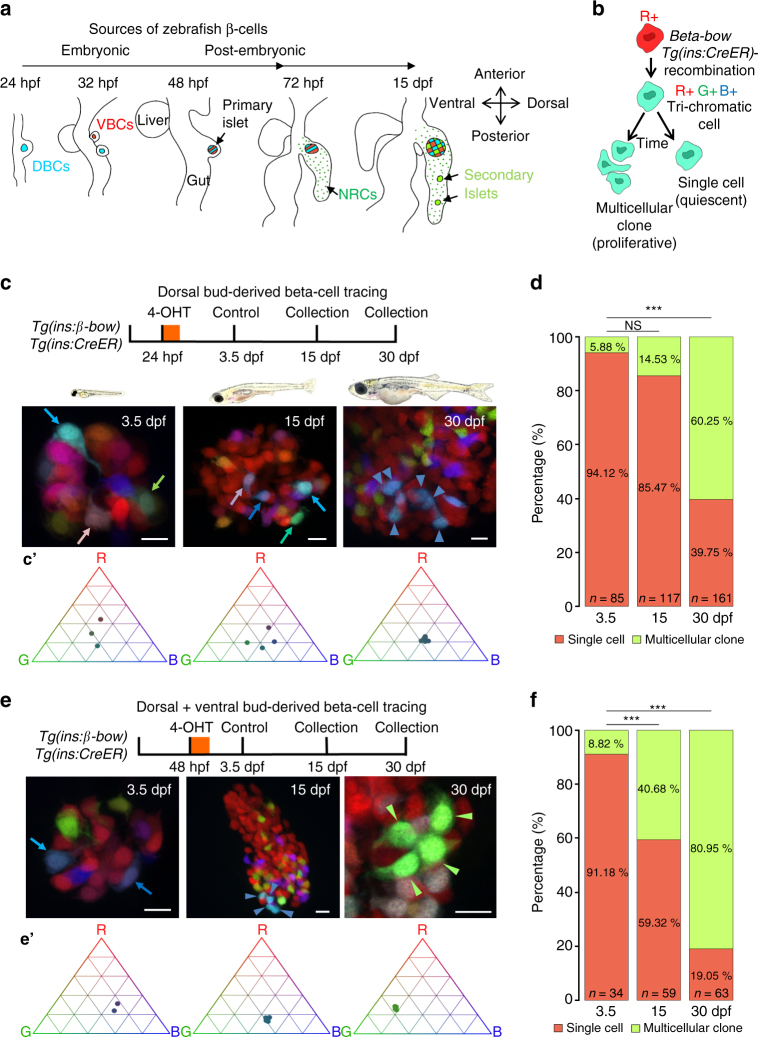
The embryonic islet contains both proliferative and long-term quiescent beta-cells. **a** Cartoon depicting the multi-lineage composition of the zebrafish primary islet. Embryonic dorsal bud-derived beta-cells (DBCs) and ventral bud-derived beta-cells (VBCs) form the embryonic primary islet. Notch-responsive cells (NRCs) are post-embryonic progenitors that make secondary islets and could contribute beta-cells to the primary islet at later stages. **b** Clonal analysis schematic. *Tg(ins:Cre-ER* ^*T2*^ *)*-recombination in *beta-bow* results in combinatorial expression of fluorescent proteins in beta-cells and unique trichromatic bar coding. Trichromatic cells can divide, forming multicellular clones or remain as single cells, indicating quiescence. **c** *Top*—DBCs were labeled in multiple colors at 24 hpf (4-OHT treatment for 6 h), and analyzed at 3.5, 15, and 30 dpf. *Bottom*—primary islets at each stage. *Arrows*—trichromatic beta-cells remaining as single cells; *arrowheads*—trichromatic cells forming multicellular clones. See also Supplementary Fig. 7 for wider views with separate channels at 30 dpf. Cartoons (*top*) show relative animal growth at each stage (to scale). **c′** Ternary plots provide visual representation of R, G, B-values for trichromatic beta-cells. Individual single cells have distinct color profiles; multicellular clones are composed of groups of cells with similar profiles. **d** Quantification showing the percentage of trichromatic cells that remain as single cells or form multicellular clones over the total number of tracked trichromatic events (*n*). At 3.5 and 15 dpf, a majority of beta-cells remain as single cells, indicating quiescence among DBCs. At 30 dpf, 60% of the beta-cells form multicellular clones (Fisher’s exact test, NS: *p* > 0.05; ***p ≤ 0.001). **e** *Top*—the combined population of dorsal and ventral bud-derived beta-cells (D + VBCs) were labeled at 48 hpf (4-OHT treatment for 3 h) and analyzed at 3.5, 15, and 30 dpf. *Bottom*—primary islets at each stage. *Arrows*—trichromatic beta-cells remaining as single cells; *arrowheads*—trichromatic beta-cells forming multicellular clones. See also Supplementary Fig. 7 for wider views at 30 dpf. **e′** Ternary plots showing the R, G, B-values for single cells and multicellular clones. **f** The proportion of multicellular clones increases significantly at both 15 and 30 dpf compared to 3.5 dpf (Fisher’s exact test, NS: *p* > 0.05; *** *p* ≤ 0.001), suggesting that proliferative beta-cells are present within the D + VBC population throughout development. Abbreviations: dpf days post-fertilization, hpf hours post-fertilization, 4-OHT 4-hydroxytamoxifen. **d**, **f** represent more than 50 recombined islets/stage from several repeats. *Scale bars*, 10 µm

To explore how beta-cells allocate growth-promoting and functional tasks, we utilized the zebrafish primary islet as a model (Fig. 1a). Because of its stereotypical positioning in the pancreas, this islet can be followed throughout embryonic and post-embryonic development^21^. During embryonic development, which refers to the developmental processes that take place until 72 h post-fertilization (hpf)^22^, beta-cells first differentiate in the dorsal pancreatic bud to form the primary islet. Additional beta-cells differentiate later in the ventral pancreatic bud and coalesce with the pre-existing beta-cells in the primary islet^21^. During the post-embryonic stages, which include the larval-to-juvenile transition that typically occurs at 4 weeks post fertilization^22^, beta-cells differentiate from post-embryonic progenitors, which line the pancreatic ducts^23^. Thus, the primary islet contains beta-cells from different lineages, potentially allowing to explore how this diversity impacts on the islet’s functional and proliferative heterogeneity^24^. Importantly, zebrafish beta-cells are required for glucose homeostasis and for organismal growth, as in mammals^25–27^.

To study beta-cell development with single-cell resolution, we developed technologies capable of monitoring the contribution of beta-cells to islet growth and function. We show that the islet harbors beta-cells from distinct temporal lineages that differ in their proliferative and functional properties. Lineage tracing to distinguish younger and older populations during islet maturation shows that the older beta-cells exhibit robust glucose responsiveness, whereas the younger beta-cells are more proliferative and less functional. While the period of islet remodeling provides a rich source of heterogeneity, we find that this heterogeneity diminishes as the islet approaches its mature state. Our study establishes a new model allowing to investigate the dynamic nature of beta-cell heterogeneity.

## Results

### Multicolor tracing reveals proliferative heterogeneity

To monitor the contribution of individual beta-cells to growth, we adapted the Brainbow methodology^2, 28^ for studying beta-cells, henceforth called *beta-bow* (Supplementary Fig. 1A and described in Methods section). *Beta-bow* allows to mark beta-cells in discrete colors and to follow their development at the single-cell level (Fig. 1b). In its default state, *beta-bow* marks beta-cells with red fluorescence. Induction of recombination using a beta-cell-specific and tamoxifen-inducible line, *Tg(ins:Cre-ER* ^*T2*^ *)*, can switch the red fluorescence of *beta-bow* to either green or blue in multiple cassettes within one genomic integration site (Supplementary Fig. 1B), producing a color palette that can specifically label beta-cells in distinct color combinations. The expression of either green or blue fluorescence together with the red color in beta-cells can arise after a single-recombination event in *beta-bow*. In contrast, the induction of trichromatic cells, which express altogether the green, blue and, red colors, requires at least two independent recombination events in the same cell, which can occur with lower probability (Supplementary Fig. 1B). Indeed, induction of recombination at 24 hpf, followed by image analysis at 3.5 dpf, revealed that the formation of trichromatic cells is rare in individual islets. On average, we observed 0.8 trichromatic events per islet, including singlets and pairs of cells consisting of two adjacent cells with the same color signature (Supplementary Fig. 1C, D). In addition, the analysis of samples at later time points (15 and 30 dpf) showed that the mean frequency of trichromatic events remained similar between stages (Supplementary Fig. 1D). Hence, to trace individual beta-cells with confidence, we focused our analysis on the trichromatic cells only, as they allow for sparse labeling of beta-cells. We developed an analysis pipeline allowing us to extract color-coded and clonal data from large number of samples in order to compensate for the rarity of the trichromatic cells (Supplementary Fig. 2).

To characterize beta-cell expansion in the primary islet during animal growth, we measured the volume of individual beta-cells marked in different colors, as well as the total volume of the islet’s beta-cells. From 3.5 to 15 days post-fertilization (dpf), the total volume of the beta-cells increased by three-fold, and from 15 to 30 dpf, by 17-fold, giving a total increase of 50-fold (Supplementary Fig. 3A and 3B). Since the average volume of individual beta-cells does not increase during this period (Supplementary Fig. 3C), the expansion is not driven by cellular hypertrophy. Therefore, we investigated how individual beta-cells contribute to growth via proliferation.

To this end, we performed clonal analysis (Fig. 1b, c) of the dorsal bud-derived beta-cells (DBCs), which constitute the earliest beta-cell population in the embryonic islet^21^. To induce *beta-bow* recombination in DBCs, we treated embryos at 24 hpf with the active metabolite of Tamoxifen, 4-Hydroxytamoxifen (4-OHT), which has a very short half-life in vivo. To prevent recombination in the ventral bud-derived beta-cells (VBCs), which form after 32 hpf, we washed away the 4-OHT at 30 hpf. To confirm that washing away the 4-OHT effectively prevents recombination at later stages of development, we incubated embryos with 4-OHT from 6 to 12 hpf, 2 h before the onset of insulin expression in DBCs^29^. We did not observe trichromatic recombination of *beta-bow* even 30 days later (30 dpf), indicating that washing away the 4-OHT effectively prevented recombination (Supplementary Fig. 4A). In addition, trichromatic cells could not form in the absence of 4-OHT (Supplementary Fig. 4B).

Hence, we proceeded with the clonal analysis of DBCs using 4-OHT treatments from 24 to 30 hpf. The clonal analysis indicated that individual DBCs exhibit significant differences in their proliferative history during islet growth. At 3.5 and 15 dpf, most traced beta-cells remained as single cells (94.1 and 85.5%, respectively), indicating that a majority of DBCs remain quiescent (Fig. 1c, d; Supplementary Fig. 5A). However, at 30 dpf, the proportion of DBCs remaining as single cells was reduced to 40%, while the remainder of cells formed multicellular clones (Fig. 1c, d; Supplementary Figs. 6A and 7A). Thus, the clonal analysis indicates that a fraction (40%) of the DBCs do not divide despite the 50-fold increase in beta-cell mass until 30 dpf. Notably, the early developmental quiescence of DBCs can be reversed, as injury induced by spatially confined islet photoablation (at 48 hpf) (Supplementary Fig. 8) or treatment with the small molecule Harmine (from 3 to 5 dpf) (Supplementary Fig. 9), which can promote exit from quiescence in human beta-cells^30, 31^, both decreased the proportion of DBCs that remain as single cells, while increasing the proportion of multicellular clones. In sum, beta-cells from the same population exhibit diverse contributions to islet growth, and non-dividing cells exist irrespective of the large increase in islet size.

We noticed that while a majority (85.5%) of the DBCs do not divide from 3.5 to 15 dpf, the islet’s beta-cell mass keeps increasing by three-fold (Supplementary Fig. 3B). To help explain this deviation, we asked if the later-born, VBCs, which contribute ~30% of the islet’s beta-cells at 48 hpf^24^, proliferate during this period to help increase the mass. To this end, we performed clonal analysis in the complete embryonic population, including the dorsal and VBCs (D + VBCs) by applying 4-OHT at 48 hpf (Fig. 1e). Remarkably, unlike the DBCs, 41% of the cells in D + VBC formed multicellular clones at 15 dpf (Fig. 1e, f; Supplementary Fig. 5B). The higher proliferative contribution of these cells was also evident at 30 dpf, as the D + VBC population formed a higher proportion of larger multicellular clones (≥4 cells per clone) compared to the DBCs (Fig. 1e, 1f; Supplementary Figs. 6B and 7B). We also compared the proliferation of DBCs and the combined D + VBC population during a short but equivalent developmental window. For this purpose, we used the CRE-reporter line, *Tg(ins:Red-Stop-Green)*, which switches from mCherry to GFP expression in beta-cells upon *Tg(ins:Cre-ER* ^*T2*^ *)*-mediated recombination. Using this system, we labeled genetically the DBCs or the D + VBCs with GFP expression at 24 and 48 hpf, respectively. In both cases, we performed 5-ethynyl-2’- (EdU) incorporation analysis from 3 to 5 dpf in order to mark all cells that replicate during this period. Consistent with the clonal analyses using *beta-bow*, only 3.1 ± 0.8% (*n* = 70 cells in five islets) of the traced DBCs were EdU-positive at 5 dpf. In contrast, during the same interval, 17 ± 0.5% (*n* = 173 cells in 7 islets) of the traced cells in the combined D + VBC population became EdU-positive (Supplementary Fig. 10). These analyses confirm that a higher proportion of proliferating beta-cells are present in the combined D + VBC population, as compared to the DBC population alone. Although we cannot label the VBCs specifically, our results strongly suggest that the higher proliferation in the combined D + VBC population comes from the contribution of VBCs. These proliferating cells could in part account for the observed increase in beta-cell mass from 3 to 15 dpf.

### Embryonic and post-embryonic lineages build the islet

**Fig. 2:**
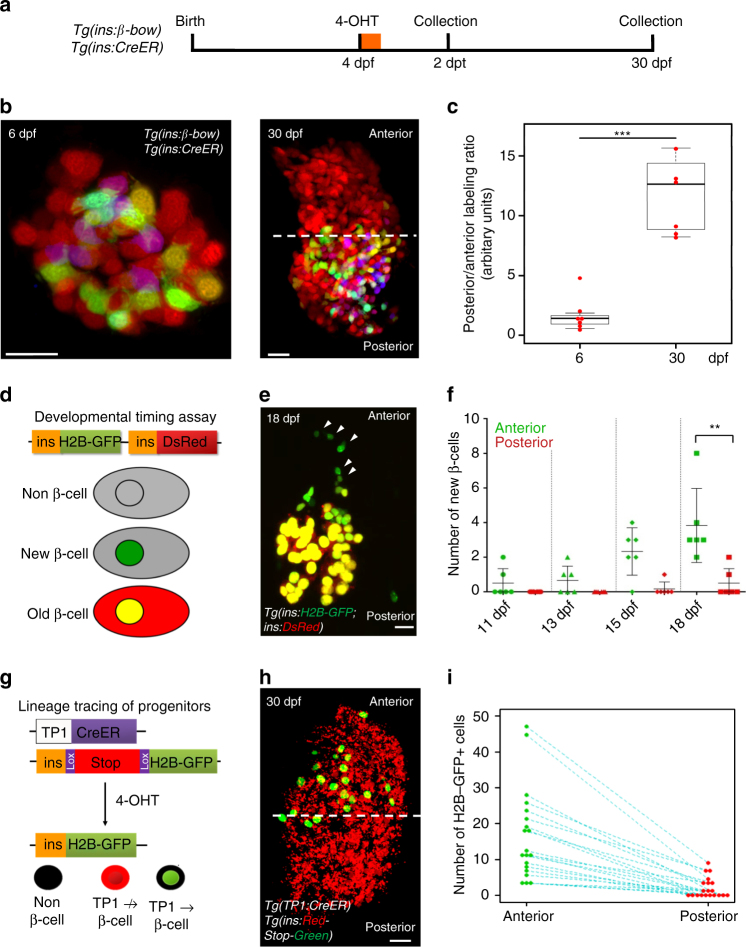
Post-embryonic and embryonic beta-cell lineages coexist in the islet’s anterior and posterior regions. **a** Beta-cells were labeled using *beta-bow* at 4 dpf and traced until 6 or 30 dpf to determine the localization of their progeny inside the islet. **b** Primary islets at 6 and 30 dpf. Multicolor beta-cells exhibit uniform distribution at 6 dpf. At 30 dpf, the multicolor cells localize within the islet’s posterior half. The anterior half contains un-recombined cells exhibiting *red* fluorescence. *Dashed line* marks the center of the islet’s A/P axis. **c** Tukey style boxplot showing quantification of the posterior/anterior labeling ratio, which measures the YFP and CFP florescence intensities in the posterior vs. the anterior half of the islet in order to determine the relative abundance of multicolor cells in each half. The 6 dpf islets exhibit similar distributions, while the 30 dpf islets contain more multicolor cells within the posterior half (*n* = 6 islets at each stage) (unpaired two-tailed *t*-test, *p* ≤ 0.001). **d** The developmental timing assay consists of two fluorescent reporters, EGFP, and DsRed, both expressed under the insulin promoter. The relatively faster maturation of GFP compared to DsRED allows to transiently mark the recently formed beta-cells with only *green* fluorescence, and to distinguish them from older beta-cells, which express both colors. **e** A primary islet from *Tg(ins:H2B-GFP; ins:DsRed)* animals at 18 dpf. A streak of recently formed cells is present near the islet’s anterior side (*arrowheads*). **f** Time course of recently differentiated beta-cells from 11–18 dpf, showing an increase in their numbers preferentially in proximity to the islet’s anterior region at 18 dpf (*n* = 6 islets per time point) (paired two-tailed *t*-test, **p ≤ 0.01). Plot shows the ﻿mean ± S.D. **g** The *Tp1* promoter drives Cre-ER^T2^-expression in NRCs. CRE-mediated recombination excises the floxed mCherry in NRCs. NRCs that undergo successful recombination and subsequent beta-cell differentiation, activate insulin:H2B-GFP-expression. **h** *Tg(Tp1:Cre-ER* ^*T2*^ *); Tg(ins:Red-Stop-Green)* animals were treated with 4-OHT at 4 dpf. Beta-cells differentiating from NRCs exhibit H2B-EGFP expression at 30 dpf. *Dashed line* marks the center of the islet’s anterior–posterior axis. Note that *Tg(Tp1:Cre-ER* ^*T2*^ *)* can mark ~30% of the NRCs, hence, the beta-cells that differentiate from NRCs are under-represented. **i** Number of H2B-EGFP-positive beta-cells within the anterior and posterior halves of individual islets (*blue dotted line*) at 30 dpf. All islets show an anterior bias in beta-cell differentiation (*n* = 20 islets). *Scale bars*, 20 µm

The proliferation of the embryonic beta-cells might not be the only source of islet growth, as post-embryonic beta-cell differentiation could also play a role. To test if neogenesis of beta-cells contributes to islet growth, we used *beta-bow* to label the pre-existing beta-cells in multiple colors at 4 dpf and traced them until 30 dpf. If new beta-cells differentiate post-recombination, these cells would express only red fluorescence, which does not require *beta-bow* recombination. The pre-existing beta-cells were uniformly labeled with multiple colors at 6 dpf, 2 days after recombination (Fig. 2a–c). Strikingly, at 30 dpf, the multicolor cells occupied preferentially the posterior regions of the islet, while the anterior regions contained beta-cells exhibiting the default red fluorescence of *beta-bow* (Fig. 2a–c). A similar topological bias was observed when the pre-existing beta-cells were traced from 2.5 to 30 dpf or even until adulthood (90 dpf) (Supplementary Fig. 11A, B), suggesting a process of extensive addition of de novo beta-cells to the islet’s anterior regions, whereas the pre-existing beta-cells are allocated to the posterior region.

To determine what proportion of the embryonic beta-cells localize within the islet’s anterior and posterior regions at 30 dpf, we used *Tg(ins:Cre-ER* ^*T2*^ *)* and the CRE-reporter line, *Tg(ins:Red-Stop-Green)*, to label the embryonic beta-cells genetically with GFP at 3 dpf (Supplementary Fig. 11C). At 30 dpf, only 13% of the traced GFP-positive cells were located within the islet’s anterior half (Supplementary Fig. 11C, D), suggesting a drastically lower contribution of embryonic beta-cells to the islet’s anterior region, as compared to the posterior.

Furthermore, we followed the formation of newborn beta-cells. To do so, we applied the developmental timing assay, which consists of two fluorescent reporters, EGFP and DsRed, both expressed under the insulin promoter. The relatively faster maturation of GFP compared to DsRED allows to transiently mark the recently formed beta-cells with only *green* fluorescence, and to distinguish them from older beta-cells, which express both colors^32^. We found that between 11 and 18 dpf, newborn beta-cells were forming preferentially in proximity to the islet’s anterior side, with their numbers increasing at 15 and 18 dpf (Fig. 2e, f).

We reasoned that progenitors that differentiate into beta-cells in proximity to the islet’s anterior regions could underlie this asymmetric formation of new beta-cells. We focused our attention on the Notch-responsive cells (NRCs), which comprise a post-embryonic progenitor pool lining the pancreatic ducts^23, 32^. NRCs were specifically marked using *Tg(Tp1:H2B-mCherry)*. Due to the slow turnover of histone-tagged proteins, the *Tg(Tp1:*H2B-mCherry*)*-positive progenitors retain H2B-mCherry fluorescence upon differentiating into beta-cells, while at the same time they activate the insulin promoter^32^. This short-term lineage tracing of the Notch-responsive progenitors showed that at 18 dpf, the anterior side of the islet contained several folds more beta-cells that differentiated from NRCs (beta-cells that exhibit H2B-mCherry-fluorescnce) compared to the posterior (Supplementary Fig. 12A, B). Long-term genetic lineage tracing of these progenitors (from 4 to 30 dpf) using the tamoxifen-inducible line *Tg(Tp1:Cre-ER* ^*T2*^ *)* and the CRE-reporter line, *Tg(ins:Red-Stop-Green)* confirmed that they contribute preferentially to the beta-cell mass in the anterior half of the islet (Fig. 2g–i). This bias was not due to preferential labeling of NRCs on the anterior side of the pancreas, as NRCs exhibited uniform genetic labeling all around the islet (Supplementary Fig. 12C) without leaky recombination (Supplementary Fig. 12D). Moreover, by histology, we found that at 18 dpf, numerous beta-cells formed at the junction where the *Tg(Tp1:*H2B-mCherry)-positive duct cells connect to the extra-pancreatic duct, an anatomical region in proximity to the anterior tip of the islet (Supplementary Fig. 13A). Beta-cell forming in this region seem to transit between acinar cells as individual cells or collectively, as they coalesce with the anterior side of the primary islet (Supplementary Fig. 13B, C).

We performed two additional experiments to test if the post-embryonic progenitors indeed contribute beta-cells predominantly to the islet’s anterior region. First, we ablated conditionally the beta-cells from 4 to 5 dpf using the nitroreductase system^33^, and then analyzed the pattern of regeneration (Supplementary Fig. 14A, B), which relies on beta-cell neogenesis but not on proliferation of beta-cell escaping ablation^34^. We predicted that regeneration of the beta-cell mass would occur faster in the islet’s anterior region, given the more extensive contribution from progenitors. Strikingly, at 14 days post-ablation (dpa) (18 dpf), complete regeneration was evident only in the anterior region of the islet (Supplementary Fig. 14C–E), whereas regeneration of the posterior region took longer, and was evident by 26 dpa (30 dpf) (Supplementary Fig. 14F, G). Second, we asked if increasing progenitor differentiation would also lead to preferential addition of beta-cells on the anterior side. To this end, we inhibited pharmacologically the Notch and the Nf-kB signaling pathways, which can inhibit NRC-differentiation^23, 32, 35^. The developmental timing assay showed that pharmacological inhibition of Notch- or Nf-kB signaling (using LY411575 and ethyl pyruvate (EP), respectively) enhanced the number of de novo beta-cells, as compared to controls in the anterior half of the primary islet but not the posterior (Supplementary Fig. 15).

We conclude that during post-embryonic growth, a majority of the islet’s anterior beta-cell mass is produced by the differentiation of post-embryonic progenitors. Thus, similar to an “hourglass” model for islet growth (Supplementary Fig. 16), the progeny of the “older” embryonic beta-cells ends up within the posterior half of the islet. Some of these beta-cells are quiescent, while the rest of them divide and contribute to increasing the beta-cell mass. As development proceeds, “younger” beta-cells differentiate from the post-embryonic progenitors and accumulate preferentially on the islet’s anterior side.

### The embryonic beta-cells are glucose-responsive

The presence of this diverse collection of coexisting cells allowed us to investigate whether beta-cells with different developmental histories exhibit different functional properties. As a measure of beta-cell functionality, we developed a reporter system that can measure glucose responsiveness of beta-cells by monitoring the glucose-induced influx of calcium, which triggers insulin release from beta-cells. To monitor intracellular calcium levels, we generated a new transgenic line expressing the genetically encoded calcium indicator GCaMP6s^36^ in beta-cells, *Tg(ins:GCaMP6s)* (Supplementary Fig. 17). In this reporter, glucose stimulation leads to an influx of calcium in beta-cells. Intracellular calcium binds GCaMP6s, leading to conformational change and an increase in the emission of green fluorescence.

**Fig. 3:**
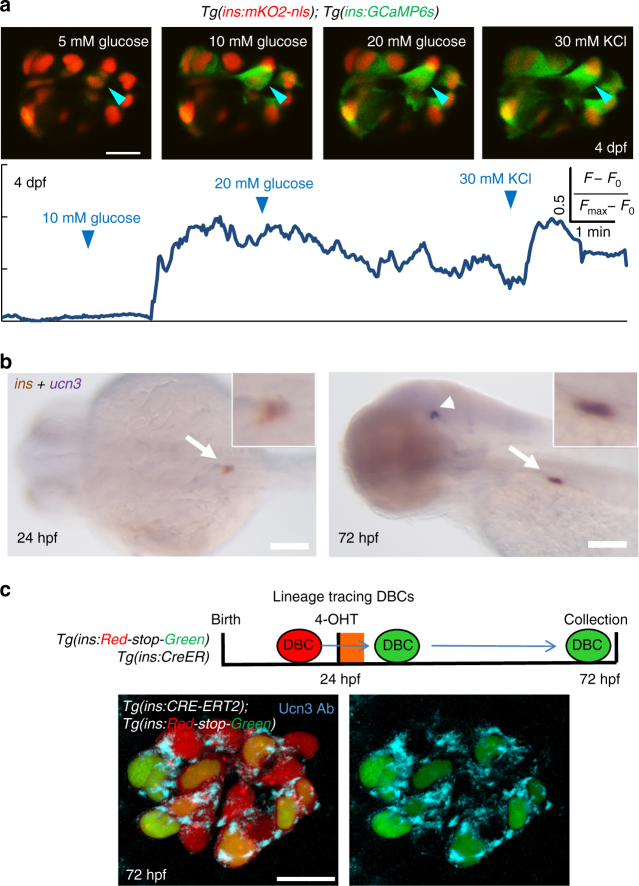
Beta-cells in the primary islet become functional during early development. **a** Glucose responsiveness of larval beta-cells at 4 dpf. *Top*—islets from *Tg(ins:GCaMP6s)*; *Tg(ins:mKO2-nls)* animals were mounted ex vivo. Beta-cells (*red nuclei*) were stimulated with a glucose ramp consisting of sequential incubation with 5 (basal), 10, and 20 mM d-glucose, and depolarized via addition of 30 mM KCl. A representative beta-cell is marked with an *arrowhead*. *Bottom*—trace of normalized GCaMP6s-fluorescence intensity over time for the beta-cell indicated in the *top panels*. In response to glucose stimulation, the cell showed strong fluorescence; indicating glucose-stimulated calcium influx. The fluorescence reached its peak after addition of KCl. 60 ± 3% of the recorded beta-cells exhibited glucose-induced calcium influx upon sequential incubation with 10 and 20 mM glucose (*n* = 36 cells in five islets) but not at basal concentrations. **b** Whole-mount, double in situ hybridization for insulin (*brown*) and *ucn3* (*purple*). At 24 hpf, *ucn3* transcripts were not detectable in the embryonic islet (*arrow*) (enlarged *inset*). At 72 hpf (3 dpf), beta-cells exhibit double positivity for insulin and *ucn3* (*arrow*) (enlarged *inset*). *Arrowheads* point to *ucn3*-expressing neurons at 72 hpf, as shown previously^38^. See also Supplementary Fig. 18 for single *ucn3* in situ hybridization at 3 and 5 dpf. **c** The *ins* promoter drives Cre-ER^T2^ expression in beta-cells. CRE-mediated recombination excises the floxed mCherry in *Tg(ins:Red-Stop-Green)*. Beta-cells that undergo successful recombination activate insulin:H2B-GFP-expression. To label DBCs specifically, 4-OHT was applied from 24 to 30 hpf. At 72 hpf, a majority of the traced beta-cells (H2B-GFP-positive) showed Ucn3-immunofluorescence (confocal projection) (*n* = 5 islets). *Scale bars* in **a**, **c**, 10 µm; 1 mm in **b**

Using this system, we first characterized the glucose-induced calcium influx in dissected islets from early larvae (4 dpf), in which the islet is mainly composed of the embryonic beta-cell lineage. As soon as 72 h after the islet had formed, the beta-cells exhibited calcium influx upon sequential stimulation with 10 and 20 mM glucose, which culminated after beta-cell depolarization using potassium chloride (KCl) (Fig. 3a) (Supplementary Movie 1). On average, 60 ± 3% (*n* = 5 islets) of the beta-cells responded to the glucose ramp but not at basal glucose (5 mM), indicating that a majority of the cells are glucose-responsive.

To characterize further the development of beta-cell functionality in zebrafish, we examined the expression of the zebrafish ortholog^37^ of the mammalian marker of beta-cell maturity, Urocortin 3 (Ucn3)^38, 39^. Ucn3 is a sensitive marker of functional maturation in mouse beta-cells, as well as in beta-cells derived from human embryonic stem cells^38, 39^. By performing whole-mount in situ hybridization (WISH) at 3 and 5 dpf, we observed that *ucn3* transcripts were expressed in the zebrafish islet as early as 3 dpf (Supplementary Fig. 18A, B). Furthermore, double WISH for insulin and *ucn3* showed that *ucn3* transcripts were not detectable at 24 hpf, soon after the embryonic beta-cells differentiate, whereas at 72 hpf (3 dpf), beta-cells exhibited double positivity for insulin and *ucn3* (Fig. 3b; Supplementary Fig. 18C, D).

To determine if the earliest population of embryonic beta-cells contributes to the pool of Ucn3-positive beta-cells, we labeled specifically the DBCs with GFP expression at 24 hpf using *Tg(ins:CRE-ER* ^*T2*^ *)* and the CRE-reporter line, *Tg(ins:Red-Stop-Green)* and traced them until 72 hpf. Indeed, at 72 hpf, a majority of the traced (GFP-positive) DBCs showed Ucn3 immunofluorescence (Fig. 3c). In agreement with the *ucn3* WISH, there was no observable Ucn3 immunoreactivity in beta-cells at 24 hpf, as compared to 72 hpf (Supplementary Fig. 18C). From these analyses, we infer that the embryonic beta-cells start expressing Ucn3 during the late embryonic stages and are capable of responding to glucose stimulation as early as 4 dpf.

### Young and old islet cells differ in function

**Fig. 4:**
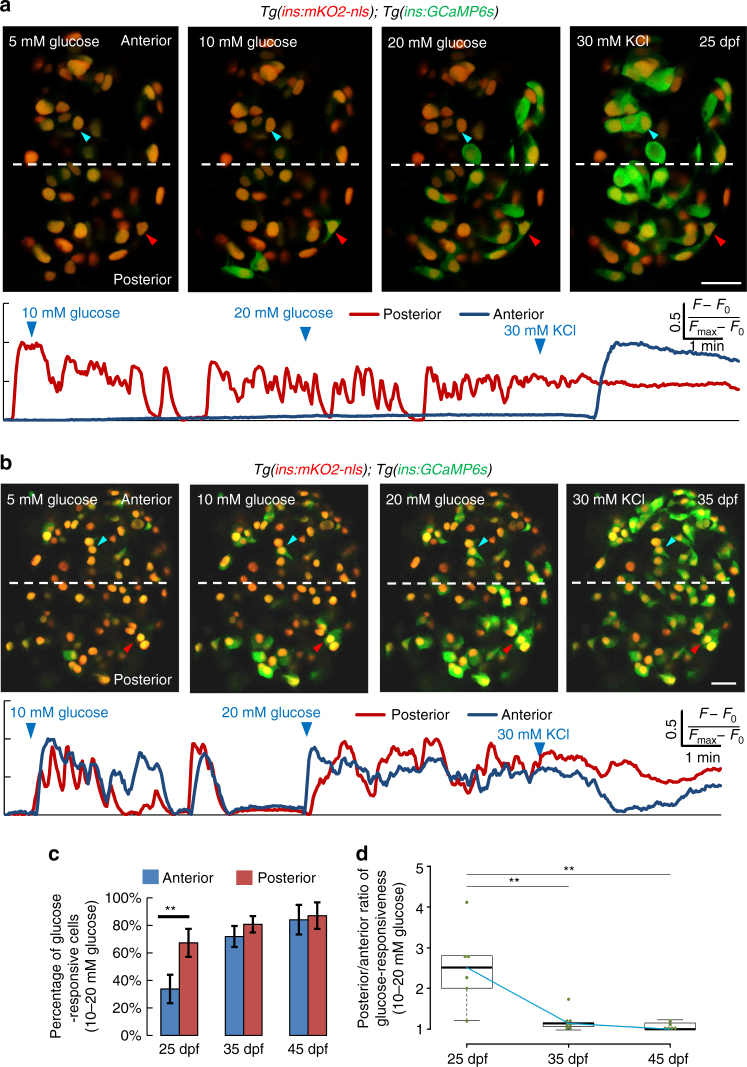
The islet’s anterior and posterior cells exhibit temporal differences in glucose-responsiveness during post-embryonic development. **a** *Top*—ex vivo live imaging of islets from 25 dpf *Tg(ins:GCaMP6s);Tg(ins:mKO2-nls)* animals. Beta-cells (*red nuclei*) were stimulated with a glucose ramp consisting of sequential incubation with 5 (basal), 10, and 20 mM d-glucose, and depolarized via addition of 30 mM KCl. A beta-cell in the islet’s posterior regions (*red arrowhead*) exhibits increasing GCaMP6s-fluorescence in response to glucose. The anterior cell (*blue arrowhead*) only responds after KCl addition. More beta-cells in the posterior region respond to glucose, as compared to the anterior. *Bottom*—trace of normalized GCaMP6s-fluorescence intensity over time for each of the two cells indicated in the *top panels* (posterior cell—*red trace*; anterior cell—*blue trace*). **b** *Top*—ex vivo imaging of islets at 35 dpf as in **a**. Beta-cells in the islet’s anterior (*blue arrowhead*) and posterior regions (*red arrowhead*) exhibit increasing GCaMP6s fluorescence in response to glucose. *Bottom*—trace of normalized GCaMP6s fluorescence intensity for each of the two cells indicated in the *top panels* (posterior cell—*red trace*; anterior cell—*blue trace*). **c** Quantification showing the mean percentage of beta-cells that respond upon sequential stimulation with 10 and 20 mM glucose in the islet’s anterior and posterior halves. At 25 dpf, more beta-cells respond to glucose in the posterior half, as compared to the anterior (paired two-tailed *t*-test, **p ≤ 0.01). At 35 and 45 dpf, both halves show similar percentage of glucose-responsive cells (*n*_25 dpf_ = 90 cells in the anterior and 121 cells in the posterior region from 6 islets; *n*_35 dpf_ = 133 cells in the anterior and 162 cells in posterior region from 7 islets; *n*_45 dpf_ = 159 cells in the anterior and 233 cells in posterior region from 6 islets). *Error bars* = S.E.M. **d** Tukey style boxplot showing the posterior/anterior ratio of glucose responsiveness at 25, 35, and 45 dpf. *Blue line* connects the medians at each stage. The differences in glucose responsiveness between the islet’s anterior and posterior regions diminish with increasing age, as the ratio is close to one at 45 dpf. (Kruskal–Wallis test, *p*-value = 0.0039; Wilcox test, **p ≤ 0.01). *Scale bars*, 20 µm

We asked how the addition of younger beta-cells impacts on the functional properties of the islet. We focused on the period of rapid islet expansion, when beta-cell differentiation from post-embryonic progenitors contributes significantly to the beta-cell mass in the islet’s anterior regions, whereas the older, embryonically derived beta-cells occupy preferentially the islet’s posterior regions (Fig. 2). To assess if this heterogeneity impacts on the functional properties of the islet, we compared the glucose responsiveness of beta-cells in the anterior and posterior halves of islets from 25 dpf animals. Remarkably, whereas 67 ± 10% of the beta-cells in the posterior half of the islet exhibited calcium oscillations upon sequential stimulation with 10 and 20 mM glucose, the same stimulation triggered responses in only 34 ± 10% of the beta-cells in the anterior region (Fig. 4a and c; Supplementary Movie 2) (*n* = 6 islets). These differences led to a significant inequality in glucose responsiveness along the islet’s anterior–posterior axis (Fig. 4d). In agreement, Ucn3 immunofluorescence marked the insulin-positive cells throughout the posterior but not the anterior regions of the islets (Supplementary Fig. 19), suggesting that the younger beta-cells in the anterior regions exhibit immature characteristics. Importantly, GCAMP imaging of islets at later stages showed that at 35 and 45 dpf, the differences in glucose responsiveness between the two halves of the islet were progressively equalizing (Fig. 4b, c, d; Supplementary Fig. 20) (Supplementary Movies 3 and 4). These results suggest that the younger beta-cells, which occupy preferentially the islet’s anterior regions, exhibit lower glucose responsiveness compared to the older beta-cell in the posterior. These differences diminish with increasing developmental time.

**Fig. 5:**
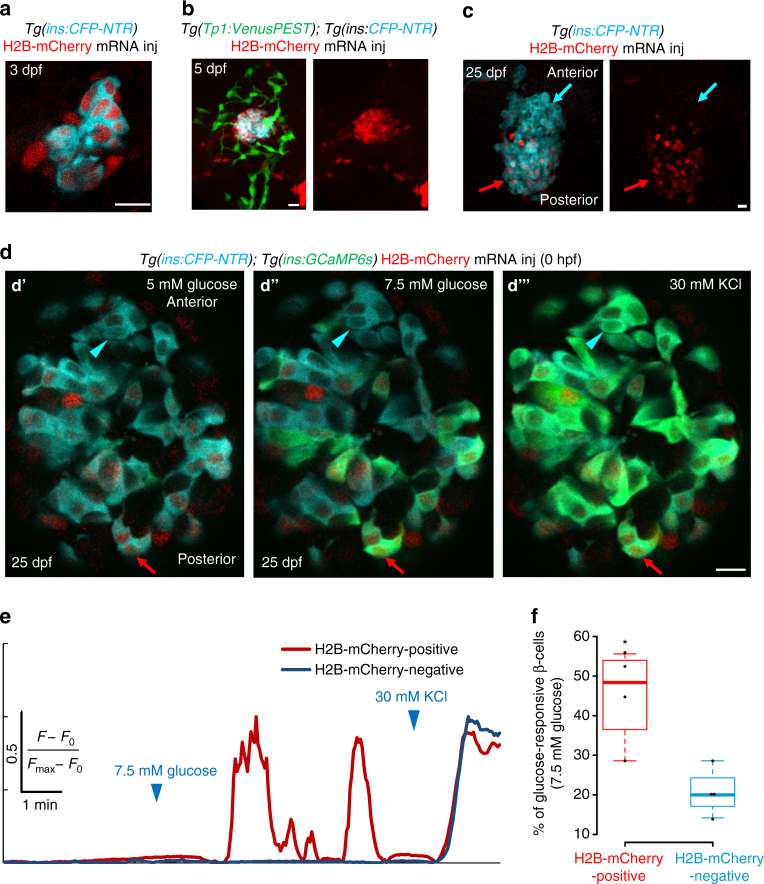
The embryonic beta-cells are highly glucose-responsive compared to beta-cells differentiating from post-embryonic progenitors. **a** Primary islets from *Tg(ins:CFP-NTR); Tg(GCaMP6s)* animals injected with H2B-mCherry mRNA at the one-cell stage and traced until 3 dpf. Beta-cells (*blue*) show uniform expression of H2B-mCherry (*red*) (single confocal plane). **b** Primary islets from *Tg(ins:CFP-NTR); Tg(Tp1:VenusPEST)* animals injected with H2B-mCherry mRNA at the one-cell stage and traced until 5 dpf. Beta-cells (*blue*) show strong expression of H2B-mCherry (*red*), whereas the post-embryonic progenitors (*green*) are H2B-mCherry-negative (confocal projection). **c** Primary islets from *Tg(ins:CFP-NTR); Tg(GCaMP6s)* animals injected with H2B-mCherry mRNA at the one-cell stage and traced until 25 dpf. The H2B-mCherry-negative beta-cells localize preferentially within the anterior regions of the islet (*blue arrow*), whereas the H2B-mCherry-positive cells occupy the posterior (*red arrow*) (confocal projection). **d** Ex vivo live imaging of islets from *Tg(ins:GCaMP6s); Tg(ins:CFP-NTR)* animals at 25 dpf injected with H2B-mCherry mRNA at the one-cell stage. Beta-cells (*blue*) were stimulated with 5 (basal) **d′** and 7.5 mM d-glucose **d′′** followed by depolarization via addition of 30 mM KCl **d′′′** while monitoring GCAMP6s-fluorescence (*green*). A *red arrow* indicates an H2B-mCherry-positive beta-cell, whereas the *blue arrowhead* indicates an H2B-mCherry-negative beta-cell. **e** Normalized GCaMP6s fluorescence intensity trace. The H2B-mCherry-positive cell (*red trace*, *red arrow* in **c**) exhibits oscillating GCaMP6s-fluorescence in response to glucose, while the H2B-mCherry-negative cell (*blue trace*, *blue arrowhead* in **c**) only responds to depolarization with KCl. **f** Tukey style boxplot showing that a higher proportion of the H2B-mCherry-positive beta-cells respond to glucose, as compared to the H2B-mCherry-negative beta-cells. A cell was considered as H2B-mCherry-negative if the mean fluorescence intensity was similar to background (*n* = 46 H2B-mCherry-positive and 44 H2B-mCherry-negative cells from four islets) (unpaired two-tailed *t*-test, *p < 0.05). *Scale bars*, 10 µm

Next, we combined calcium imaging with the histone retention assay, which would allow to distinguish the older from the younger beta-cells in the islet based on the high perdurance of fluorescently tagged histones^24^. In this assay, messenger RNA (mRNA) encoding the fluorescently tagged histone H2B-mCherry is injected into the one-cell stage embryo. Since each cell division exponentially dilutes the fluorescently tagged histone, beta-cells differentiating earlier in organogenesis would exhibit stronger signal, whereas cells derived later would exhibit much weaker signal due to the continuous proliferation of their progenitors prior to differentiation. By injecting high concentrations of mRNA encoding H2B-mCherry, we labeled the embryonic beta-cells with nearly uniform levels of H2B-mCherry at 3 dpf (Fig. 5a). In contrast, the post-embryonic progenitors, which undergo extensive amplification early on^40^, fully diluted the histone label as early as 5 dpf (Fig. 5b), indicating that de novo beta-cells would be H2B-mCherry-negative. At 25 dpf, the islet’s posterior regions contained H2B-mCherry-positive beta-cells retaining the label from earlier stages, whereas the H2B-mCherry-negative beta-cells preferentially localized within the islet’s anterior regions (Fig. 5c, d). This pattern is consistent with our results showing that the post-embryonic progenitors preferentially contribute beta-cells to the islet’s anterior region (Fig. 2). By performing calcium imaging of islets ex vivo at 25 dpf, we compared the glucose responsiveness of the H2B-mCherry-positive and H2B-mCherry-negative beta-cells, a majority of which would represent beta-cells differentiating from post-embryonic progenitors (Fig. 5d, e). To help identify highly glucose-sensitive beta-cells, we stimulated the cells with an intermediate ramp of glucose concentrations (from 5 to 7.5 mM glucose). 45 ± 12% of the H2B-mCherry-positive beta-cells exhibited glucose-induced calcium influx, while only 21 ± 6% of the H2B-mCherry-negative beta-cells responded during the course of glucose stimulation (Fig. 5f) (Supplementary Movie 5). This experiment strengthens our conclusion that the descendants of the embryonic beta-cells represent a highly glucose-sensitive population, as compared to the younger beta-cells differentiating later on. It is important to note, however, that based on our clonal analysis (Supplementary Fig. 6), some H2B-mCherry-negative beta-cells might represent rare but more proliferative embryonic beta-cells that fully dilute the histone label. Examining the functional properties of such cells would require further investigation.

### The islet’s young and old cells differ in proliferation

**Fig. 6:**
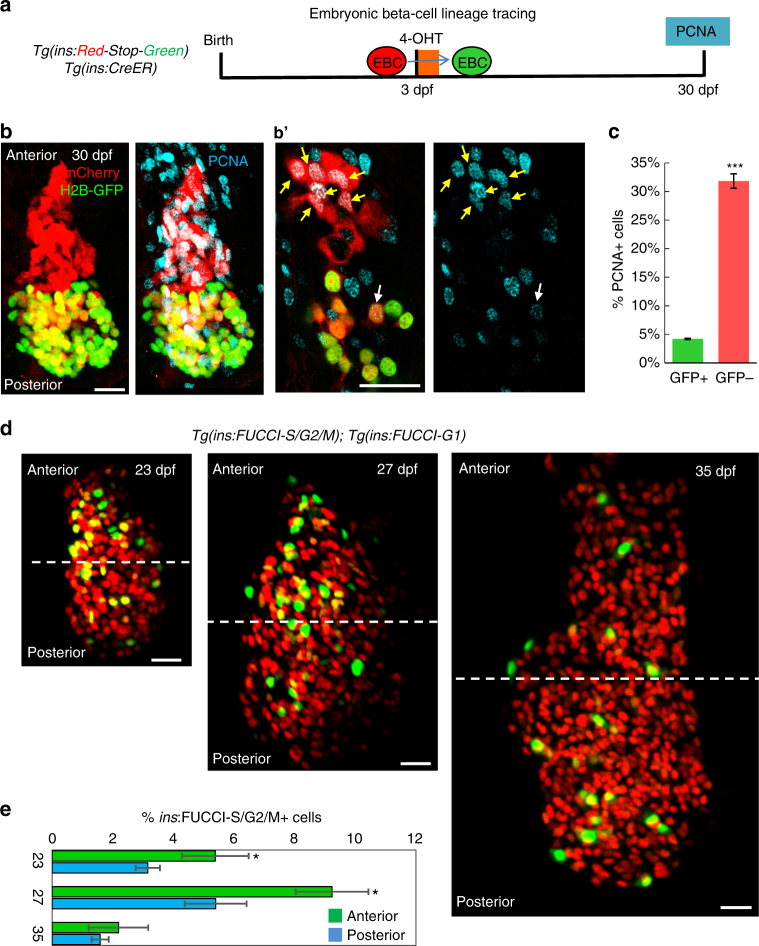
Beta-cells differentiating from post-embryonic progenitors are more proliferative compared to the embryonic beta-cells. **a** Schematic showing the labeling of the embryonic beta-cell (*EBC*) population during growth. *Tg(ins:Cre-ER* ^*T2*^ *)*—mediated recombination excises the floxed *mCherry* from *Tg(ins:Red-Stop-Green)*. Beta-cells that undergo successful recombination activate insulin:H2B-GFP expression. Recombination was induced using a 4-OHT treatment at 3 dpf. New beta-cells that differentiate post-recombination are GFP-negative and can only express mCherry. The samples were stained at 30 dpf using an antibody for PCNA, which peaks in expression during the G1/S-stages of the cell cycle. **b** Confocal projections of islets from *Tg(ins:Cre-ER* ^*T2*^ *);Tg(ins:Red-Stop-Green)* animals at 30 dpf. The GFP-positive beta-cells localize preferentially in the posterior half of the islet, whereas the anterior half is occupied by GFP-negative but mCherry-positive beta-cells. **b′** Higher magnification single planes from the cells shown in **b**. A majority of the mCherry-positive but GFP-negative beta-cells are also PCNA-positive (*yellow arrows*) whereas only one of the GFP-positive beta-cells is PCNA-positive (*white arrows*). Note that since *Tg(ins:Red-Stop-Green)* contains multiple cassettes within one genomic integration site, some of the beta-cells are both GFP and mCherry-positive due to incomplete excision of *mCherry* in all cassettes during the 4-OHT treatment. **c** Quantification showing that a higher proportion of GFP-negative beta-cells were PCNA-positive at 30 dpf compared to the GFP-positive beta-cells, indicating higher proliferative capacity of de novo beta-cells during islet growth (*n* > 50 GFP-positive and -negative cells per islet, *n* = 7 islets) (unpaired two-tailed *t*-test, ***p < 0.005). Plot shows mean ± S.E.M. **d** Confocal projections of islets from *Tg(ins:FUCCI-G1); Tg(ins:FUCCI-S/G2/M)* animals at 23, 27, and 35 dpf oriented along the anterior–posterior axis. Samples were collected ~10 h after feeding, which is important to stimulate beta-cell proliferation during the late larval and juvenile stages in zebrafish^33^. **e** Quantification of the percentage of *Tg(ins:*FUCCI-S/G2/M*)-*positive and *Tg(ins:*FUCCI-G1*)-*negative beta-cells in the anterior and posterior halves of islets from each stage. At 23 and 27 dpf, a higher proportion of proliferating beta-cells are present in the islet’s anterior half, as compared to the posterior. At 35 dpf, beta-cell proliferation is similar in both the anterior and the posterior regions. In addition, beta-cell proliferation is reduced compared to 23 and 27 dpf (*n* = 8 islets at 23 dpf; *n* = 5 islets each at 27 and 35 dpf) (paired two-tailed *t*-test, *p < 0.05). *Scale bars*, 20 µm

We asked if the younger and older beta-cells that coexist in the same islet and experience similar metabolic conditions, also differ in beta-cell proliferation. To do so, we genetically labeled the embryonic beta-cells with GFP at 3 dpf using *Tg(ins:CRE-ER* ^*T2*^ *)* and the CRE-reporter line, *Tg(ins:Red-Stop-Green)*, and traced them until 30 dpf (Fig. 6a). New beta-cells that differentiate post-embryonically would be GFP-negative, expressing the default mCherry fluorescence of *Tg(ins:Red-Stop-Green)*, which does not require recombination. To probe for differences in the proliferative potential of the younger and older beta-cells, we compared the expression of the proliferating cell nuclear antigen (PCNA) in the GFP-positive and GFP-negative beta-cells. PCNA is specifically expressed in proliferating cells throughout the cell cycle with its levels peaking in the G1/S stages^41^. This approach revealed that a much higher proportion of the younger (GFP-negative) beta-cells were PCNA-positive, as compared to the older (GFP-positive) beta-cells (Fig. 6b, c). Thus, de novo beta-cell formation provides a pool of cells with a higher proliferative potential.

**Fig. 7:**
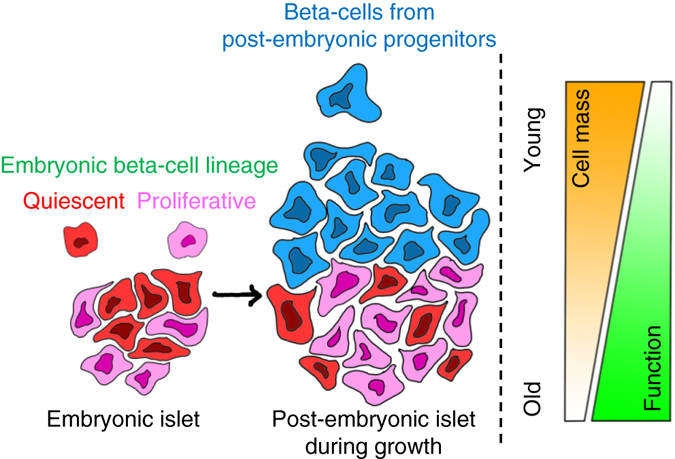
Different developmental histories of beta-cells lead to functional and proliferative heterogeneity. A model showing that beta-cells within the same islet can exhibit different behaviors based on differences in the time of their differentiation, which can shape the islet’s functional heterogeneity during maturation. The increase in the islet’s beta-cell mass involves contributions from both the proliferation of embryonic beta-cells and the addition of post-embryonic beta-cells via progenitor differentiation. New beta-cells are added preferentially to the islet’s anterior regions (*top*), whereas the embryonic beta-cells localize to the posterior (*bottom*). Thus, the islet becomes polarized in regard to cellular age. The younger beta-cells have reduced glucose responsiveness but show higher proliferation compared to the older cells. Notably, this heterogeneity dampens with increasing age, likely as the result of reduced neogenesis and maturation of the islet’s younger beta-cells

Furthermore, we analyzed if the islet’s younger (anterior) and older (posterior) regions differ in the overall beta-cell proliferation. To this end, we marked the proliferating beta-cells using the fluorescence ubiquitination cell cycle indicator (FUCCI)-reporters^42^. In this system, beta-cells that enter S-phase and progress through the cell cycle exhibit green fluorescence, whereas beta-cells in the G0/G1 stages of the cell cycle exhibit red fluorescence^32^. Quantifying the percentage of proliferating beta-cells in growing islets (at 23 and 27 dpf) revealed higher proliferation in the anterior vs. the posterior regions of the islets (Fig. 6d, e). Notably, at 35 dpf, the differences in proliferation between the islet’s anterior and the posterior regions equalized, together with a general reduction in the overall percentage of proliferating cells (Fig. 6d, e). Altogether, our experiments show that the islet’s rapid growth period involves the addition of younger, highly proliferative but less functional beta-cells, as compared to the older beta-cells that differentiate in the embryo (Fig. 7). In addition, the synchronization of proliferation and function between the anterior and posterior regions indicate a reduction in the islet’s heterogeneity with increasing age.

## Discussion

Recent studies have revealed heterogeneity in mammalian beta-cells. Thus, mature mouse beta-cells can be distinguished from the proliferative ones using the *flat-top* gene as a marker^18^. Furthermore, beta-cell hubs coordinating the islet’s function were described in mouse islets^43^, and four sub-populations of beta-cells coexist in human islets, which also differ in glucose-responsiveness^17^. An intriguing model explaining beta-cell heterogeneity posits that beta-cells can exist preferentially in proliferative or functional states and even switch states. In support of this notion, the conditionally immortalized human beta-cell line (EndoC-βH2), exhibits pronounced enhancement of beta-cell functionality, including higher insulin secretion upon undergoing cell cycle arrest^44^. Similarly, induction of senescence, a form of permanent cell cycle withdrawal, enhances beta-cell function in vivo^45^. Conversely, alleviating the work load of beta-cells by generating cells with reduced capacity for insulin production leads to higher replication rates in these cells compared to wild-type neighbors^46^. Furthermore, transcriptional analysis have shown that proliferating beta-cells reduce the levels of transcripts required for function^47^. However, understanding how beta-cells switch between proliferative and functional states remains an open question.

By applying technologies visualizing beta-cell development and function, we revealed a new model allowing to investigate how the proliferative and functional states evolve during islet maturation (Fig. 7). Our lineage tracing and pulse-chase experiments show that the increase in the islet’s beta-cell number involves contributions from both the proliferation of embryonic beta-cells and the addition of post-embryonic beta-cells (Fig. 2). Coupling this model with the visualization of calcium fluxes, revealed that younger beta-cells are less glucose responsiveness but show higher proliferation compared to the older cells (Figs. 4–6). Notably, this heterogeneity dampens with increasing developmental age, likely as a result of reduced beta-cell neogenesis and maturation of the islet’s younger beta-cells. The reduction of heterogeneity with increasing age is in agreement with a new study, which applied single-cell transcriptomics to characterize beta-cell diversity during mouse pancreas development. This approach revealed that juvenile beta-cells showed significant molecular heterogeneity reflecting distinct cell cycle phases and maturation states, whereas the adult beta-cells were comparatively homogeneous at the transcriptomic level^48^. Moreover, our study reveals the development of beta-cell functionality in the embryonic beta-cells (Fig. 3). This model could allow to investigate signaling pathways that control this process using genetic and chemical–genetic approaches afforded by the translucent zebrafish embryos. Lessons learned from development can be applied to improve the differentiation of functional human beta-cell from stem cells in vitro^49, 50^.

Recent studies suggested the presence of immature, Ucn3-negative and insulin-positive cells in the islets from donors with type 2 diabetes. There is an ongoing debate whether these cells originate from beta-cells that turn-off maturation markers due to de-differentiation^51^, or whether the immature beta-cells represent newly differentiated beta-cells^52^. It will be important to identify the source of immature cells, as this knowledge could guide the approaches for beta-cell regeneration and protection in type 2 diabetes. In this regard, a recent study showed the presence of a neogenic niche in the adult mouse pancreas that generates Ucn3-negative and glucose-unresponsive insulin-positive cells^53^. It will be interesting to determine if these younger beta-cells also exhibit higher proliferation, and whether they represent a similar population as the one we observed during zebrafish development.

In terms of de novo beta-cell formation, we found that the post-embryonic progenitors, which line the pancreatic ducts in zebrafish^23, 32, 54, 55^ provide abundantly young beta-cells preferentially to the anterior regions of the primary islet (Fig. 2), a process that also underlies asymmetric patterns of beta-cell regeneration (Supplementary Fig. 14). Potential explanations for this bias include the presence of migratory tracks in the anterior pancreas along endothelial cells or between acinar cells that direct newborn beta-cells into the primary islet. More intriguingly, studies in the Chris Wright’s group showed unequal distribution of endocrine progenitors along the pancreatic ducts in mice. Namely, the progenitors constitute an epithelial “plexus state” in which differentiation is more robust and persistent, as compared to the rest of the branched ductal arbor^56^. The cells located at the junction between the intra- and extra-pancreatic ducts, which also appear to form a “plexus”, might constitute active progenitors that preferentially supply beta-cells into the nearest side of the primary islet. Further studies will be necessary to identify the signals that increase beta-cell differentiation from these progenitors during post-embryonic development.

It is important to note that our results build on a pioneering study^24^, exploring beta-cell development using short-term tracing using the perdurance of fluorescently tagged histones. This work proposed that DBCs were non-functional and quiescent, whereas the VBCs proliferated. However, the long-term behaviors of DBCs remained a mystery, as the previously available techniques could not trace the cells during islet growth with single-cell resolution. Clearly, single-cell inspection, as recently shown for pancreatic progenitors^57^, provides a deeper understanding of cellular heterogeneity. Our single-cell analyses add novel aspects to the previous knowledge. We show that the quiescence of DBCs is not permanent, as approximately 60% of the cells form multicellular clones until 30 dpf (Supplementary Figs. 6A and 7A), indicating that proliferating DBCs clearly contribute to the beta-cell mass in the islet. Consistent with our findings, beta-cell replication was previously observed around 24 hpf, a stage when only the recently born DBCs constitute the islet^58^. Supporting the proliferative flexibility of DBCs, the small molecule Harmine or injury both triggered premature proliferation in DBCs (Supplementary Figs. 8 and 9). The ability of Harmine to stimulate proliferation in beta-cells from different species, including human beta-cells^30, 31^, suggests that certain mechanisms governing beta-cell quiescence are conserved across evolution. Finally, it will be important to determine if the quiescent and proliferative beta-cells in zebrafish also differ in functionality, as reported for quiescent and proliferative mouse beta-cells based on differential expression of *flat-top* ^18^.

In summary, we provide an example of cellular diversity in the maturing islet. Our work establishes the tools and a new developmental model to study the molecular mechanisms underlying the switch between proliferative and functional beta-cells within the same islet. Whether different beta-cell populations arise in human islets during compensatory increases in beta-cell mass remains to be addressed. Since lineage tracing is challenging in human islets, newly developed technologies, including single-cell transcriptomics^14, 59^ and mass cytometry^60^ can be applied to study the presence of different populations of human beta-cells.

## Methods

### Zebrafish strains and husbandry

Wild-type or transgenic zebrafish of the outbred AB, WIK, or a hybrid WIK/AB strain were used in all experiments. Zebrafish were raised under standard conditions at 28 °C. Animals were chosen at random for all experiments. Published transgenic strains used in this study were *Tg(ins:DsRed;ins:H2B-EGFP)* ^32^ *; Tg(Tp1:H2B-mCherry)* ^*s939*^ ^40^ *; Tg(Tp1bglob:VenusPEST)* ^*S940*^ ^40^, abbreviated *Tg(TP1:VenusPEST); TgBAC(Nkx6*.*1:eGFP)* ^61^ *; Tg(Tp1bglob:CreER* ^*T2*^ *; cryaa:CFP)* ^*s951*^ ^32^, abbreviated *Tg(Tp1:Cre-ER* ^*T2*^ *); Tg(-3*.*5ubb:loxP-EGFP-loxP-mCherry)* ^*cz1701*^ ^62^ *; Tg(insulin:loxP:mCherrySTOP:loxP:H2B-GFP)* ^*s934*^ ^63^, abbreviated *Tg(ins:Red-Stop-Green); Tg(sst:RFP)* ^*gz19*^ ^64^; *Tg(ins:CFP-NTR)* ^*s892*^ ^33^; *Tg(ins:FUCCI-G1)* ^*s948*^ ^32^; *Tg(ins:FUCCI-S/G2/M)* ^*s946*^ ^32^; ​*Tg(Tp1bglob:mCherry-CAAX*)^*s1015*^, *abbreviated Tg(Tp1:mem-mCherry)﻿﻿* ^65^. Experiments were conducted in accordance with the Animal Welfare Act and with permission of the Landesdirektion Sachsen, Germany (AZ 24–9168, TV38/2015, A12/2016, A13/2016, and all corresponding amendments).

### Construction of *Tg(ins:BB1.0L; cryaa:RFP)*

To generate the *ins:BB1.0L; cryaa:RFP* construct, an insulin promoter containing vector with multiple cloning sites (MCS) (*ins:MCS; cryaa:RFP*) was first generated. This was achieved by digesting the plasmid *ins:mAG-zGeminin; cryaa:RFP* with EcoRI/PacI and inserting dsDNA, generated by annealing two primers, harboring the sites EcoRV, NheI, NsiI, SalI, and flanked by EcoRI/PacI overhangs. *ins:MCS; cryaa:RFP* was subsequently digested with EcoRV/NsiI and the plasmid Thy1-Brainbow-1.0L^28^ (Addgene Plasmid #18725) digested with Eco53kI/NsiI to yield compatible fragments, which were ligated together to yield the final construct. The entire construct was flanked with I-SceI sites to facilitate transgenesis. Twenty founders were isolated and screened for the strength of RFP within the islet and color diversity upon inducing recombination. One line, referred as *beta-bow*, was used to perform all experiments.

### Construction of *Tg(ins:Cre-ER*^*T2*^*; cryaa:CFP)*^*s821*^

The *ins:Cre-ER* ^*T2*^ *;cryaa:Cerulean* construct was generated using the Tol2 kit system^65^. The 1.1 kb zebrafish insulin promoter was isolated by PCR, and cloned into pENTR 5′-TOPO to generate the 5’ entry clone. For the middle entry clone, *Cre-ER* ^*T2*^ cDNA amplified by PCR was cloned into pENTR-TOPO vector using the Invitrogen TOPO TA cloning kit. The resulting vectors were recombined through a Gateway LR recombination reaction with p3E-PolyA^66^, and pDestTol2pA2 introduced a *cryaa:Cerulean* transgene at BglII site.

### Construction of *Tg(ins:YFP-2A-NTR3; cryaa:RFP)*

To generate *ins:Tag-YFP-T2A-NTR3*; cryaa:mCherry construct, which expresses a triple mutant nitroreductase^66^, a *Tag-YFP-T2A-NTR3* cassette was amplified by PCR, adding the restriction sites EcoRI and PacI at its 5′ and 3′ ends. The PCR product was inserted downstream of the insulin promoter following EcoRI/PacI double-digestion to create the final plasmid. Transgenics were generated using the I-SceI meganuclease system.

### Construction of *Tg(ins:GCaMP6s; cryaa:RFP****)***

To facilitate generation of the *ins:GCaMP6s; cryaa:RFP* construct, MCS (MCS2) were first inserted downstream of the insulin promoter to yield ins:MCS2; cryaa:RFP. To do so, the plasmid ins:mAG-zGeminin; cryaa:RFP was digested with EcoRI/PacI and ligated with dsDNA generated by annealing two primers harboring the sites SpeI, BamHI, EcoRV, and EcoRI and flanked by EcoRI/PacI overhangs. Primers were designed such that EcoRI site was destroyed in the process. *ins:MCS2; cryaa:RFP* was subsequently digested with BamHI/EcoRI and the plasmid pGP-CMV-GCaMP6s^36^ (Addgene Plasmid #40753) digested with BglII/MfeI to yield compatible fragments, which were ligated together to yield the final construct. The entire construct was flanked with I-SceI sites to facilitate transgenesis.

### Construction of *Tg(ins:mKO2-Renilla-nls; cryaa:CFP)*

For the *ins:mKO2-Renilla-nls; cryaa:CFP* construct, previously published vector *ins:mKO2-zCdt1* was digested with EcoRV/PacI to excise zCdt1. The coding sequence for Renilla was amplified using primers: Eco53kI-Renilla-FP (5′-cgatcaGAGCTCCAGGTGGAGCTTCCAAGGTGTACGACCC-3′) and PacI-NLS-Renilla-RP (5′-cgatcattaattaaTTATTTCTTTTTCTTAGCTTGACCAGCTTTCTTAGTAGCAGCAGGACGCTT ACCGCCCTGCTCGTTCTTCAGCACGCG-3′). The PCR product was subsequently digested with Eco53KI/PacI and inserted into excised insulin promoter vector to yield the final construct. I-SceI sites flanked the entire cassette to facilitate transgenesis.

### 4-OHT labeling

To find the optimal treatment, which yields the appearance of trichromatic cells in *beta-bow*, we performed pilot experiments at 24 and 48 hpf with different concentration of 4-OHT followed by analysis at 3.5 dpf. The following treatment conditions were used for all tracing experiments:

D﻿BCs tracing: 1 μM﻿ 4-OHT treatment at 24 hpf for 6 hrs.

D+VBCs tracing: 2 µM 4-OHT treatment at 48 hpf for 3 hrs.

*Beta-bow* tracing at 2.5 dpf: 10 µM 4-OHT treatment for 6 h.

*Beta-bow* tracing at 4 dpf: 10 µM 4-OHT treatment for 6 h.

Beta-cell tracing at 3 dpf using *Tg(ins:Red-Stop-Green)*: 10 µM 4-OHT treatment for 6 h.

Notch-responsive cell-tracing: 10 µM 4-OHT at 4 dpf.

Treatments were performed by transferring 25 embryos or larvae to a single well of a six-well plate containing 10 ml of egg water with appropriate concentration of 4-OHT, made from a 2 mM stock in 100% ethanol. Following treatment, embryos were rinsed 3 × 30 min with egg water and placed in a fresh dish. In all 4-OHT treatments, the solution was protected from light by wrapping the containers with aluminum foil. Ethanol was used as vehicle for all control animals.

### Tissue collection

To facilitate confocal imaging of the islets, the pancreas was dissected from fish (larval) or the gut (juvenile and adults) after fixation. Fish were killed in Tricaine prior to either direct fixation or dissection of gut, and the samples immersed in 4% paraformaldehyde + 1% Triton-X for 2 days at 4 °C. The pancreas was then manually dissected and washed multiple times in PBS.

### *Beta-bow* image acquisition

To perform clonal analysis of embryonic beta-cells, beta-cell fluorescence was captured using confocal imaging of fixed whole-mount islets. Islets from *Tg(ins:BB1.0L; cryaa:RFP); Tg(ins:Cre-ER* ^*T2*^ *; cryaa:CFP)* double-transgenic fish were isolated as described above and mounted on glass slide in Fluoromount-G. Confocal slices of islets were captured using LSM 780 with 40 × 1.2 NA water immersion objective and Z-thickness of 0.51 µm. RFP, YFP, and CFP were excited using the 561, 514, and 450 nm laser lines, respectively^67^, and rendered in Red (R), Green (G), and Blue (B) channels. Channels were acquired sequentially. Images were imported into ImageJ for further processing.

### Volume estimation

For extraction of volumetric metadata from *beta-bow* images, the images were converted to 8-bit and the volume was measured using the “3D Objects Counter” plugin (http://imagej.net/3D_Objects_Counter)^68^. The plugin performs a threshold to isolate the labeled region and counts the number of labeled regions in the stack, along with providing their respective volume. For estimating total beta-cell volume, the 8-bit image was generated from the R, G, B overlay to include the entire beta-cell population within the islet. The volumes of all identified regions, encompassing the entire beta-cell mass was added to yield an estimate of the total volume. The complete process was automated to process an image folder in ImageJ macro “Process_Folder_vol.ijm”. For estimating single-beta-cell volume, the 8-bit image was generated from the Green and Blue channels only, while ignoring the Red channel. Use of only the Green and Blue channels allowed analysis of individual cells that had distinct labeling from their neighbors. Adjacent cells with similar color profiles could not be successfully separated, and thus were excluded from the analysis. The complete process was automated using the ImageJ macro “Process_Folder_Single_cell_vol.ijm”. To validate our volume estimates, we evaluated an estimate of beta-cell number within an islet and compared it to a manual count of beta-cells. For this, we randomly selected five samples each from 3.5 and 15 dpf. Beta-cell number within an islet was estimated as a ratio of total volume to the mean of single-cell volume at that stage. Beta-cells within the same islet were manually counted in a blind setup. Comparison between the two yielded less than 10% error in the estimate.

### Estimation of background recombination frequency

Untreated *beta-bow* samples were imaged at 30 dpf to estimate leaky recombination. Two islets out of 10 harbored two dichromatic cells each, indicating four leaky recombination events. An estimate of number of beta-cells at 30 dpf was made by dividing mean volume with mean single-cell volume; giving 1,51,337.80/184.70 ≈ 820 cells. The probability for single-recombination events was estimated to be 4/(820 × 10) ≈ 0.0005. The probability for appearance of a trichromatic cell without induction (requiring two-recombination events) = 2.5E−7.

### Clonal analysis

For extraction of beta-cell color signatures, fluorescent intensities corresponding to the three channels were extracted from within the nuclear region of the labeled cells, and normalized. A semi-supervised ImageJ interface (“betabow.ijm”) was developed that segments the region of interest (ROI), generates a color histogram and extracts the histogram and location statistics. The interface outputs the mean and the standard deviation for RGB channels, along with the *X*, *Y*, and *Z*- co-ordinates of the cell. The interface was used to manually select the nuclei of labeled cells in individual planes of the confocal stack, along with three unlabeled cells. The mean green and blue intensities from unlabeled cells were averaged and used for background subtraction. Background subtracted RGB values for each labeled cell were normalized by transforming them into percentages as follows: *C*_normalized_ = *C*_mean_/(*R*_mean_ + *G*_mean_ + *B*_mean_) × 100; where C is R, G, or B. (RGB)_normalized_ defines the color signature of a particular cell.

For visual representation of color signatures of beta-cells within an islet, the normalized values were plotted onto a ternary plot^69^. A ternary plot consists of an equilateral triangle, in which the vertices represent one particular dimension; a single color in this case; while the sides travel between the two adjacent dimensions. A point on the side of the triangle would only have two colors, while points toward the middle would contain all three colors. Points, or beta-cells, overlapping or adjacent to each other would share similar color profiles, and might therefore be considered as a clone. Mapping of beta-cells color signature onto a ternary plot allowed fast estimation of clones within an islet.

To statistically test clonality within the samples, we utilized “Equivalence Acceptance Criteria” by performing “Two one-sided test procedure (TOST)” on the normalized RGB values. TOST, with the null hypothesis that the two observations are not equivalent, utilizes confidence intervals based on mean and standard deviation of RGB values for calculating similarity between two observations. A macro “Pairwise_Equivalence.ijm” was developed, which generates all possible pairs between cells from which the color information was extracted. It then utilizes the mean and standard deviation values to apply TOST with *p* < 0.05 to each pair sequentially, and outputs pairs which pass the test successfully, which are then considered to be part of single clone. If a cell falls in more than one equivalent pairs, all equivalent cells are considered to be within one clone. The number of clones within an islet were recorded, along with the number of beta-cells within each clone. Application of TOST on intensity distribution allowed for clustering of beta-cells into clones.

To confidently perform clonal analysis on our samples, we restricted our analysis to triple-positive cells. To ascertain the presence of all three colors within a cell, the mean of fluorescent intensity was tested to be statistically different from zero. To do so with *p* < 0.01, which corresponds to a distance of 2.58 standard deviation (sd) from the mean, the following formulation was used:

1 $${C_{{\rm{mean}}}}- 2.58*{C_{{\rm{sd}}}}  >\, 0,\,{\rm{where}}\,C\,{\rm{is}}\,{\rm{R}},\,{\rm{G,}}\,{\rm{and}}\,{\rm{B}}.$$

A cell which satisfies the criteria for all three channels was considered as triple-positive, and carried forward for clustering into clones. Clonal analysis restricted to triple-positive cells was applied to all 3.5, 15, and 30 dpf samples, allowing tracing of beta-cells during fish growth.

### ImageJ macros package

All macros mentioned above along with plugins, a representative data set, and detailed instructions are available as a downloadable package from github (https://github.com/sumeetpalsingh/Beta-bow.git).

### Comparison along the anterior/posterior (A/P) axis

For all comparisons between the anterior and posterior region of the primary islet, the islet was equally divided into two halves along the A/P axis using maximum intensity projection generated from the confocal stack in ImageJ. To divide the islet into two halves, the islet’s center was first located as the middle of the A/P axis drawn by placing a straight line connecting the anterior tip to the posterior one. The center was used to draw another line perpendicular to the A/P axis, which served as the divider.

To extract fluorescence intensity data from the two halves, the islet area on each side of the divider was outlined manually and added as a “Region of Interest” (ROI). The total fluorescence intensity and the area were measured using “Integrated density” and “Area”, respectively, on the ROIs. For *beta-bow*-based fluorescence quantification, the islet was divided using all three channels, but measurements were made using the blue and green signal only. For NTR-based regeneration experiments, the *sst:*RFP signal was used for islet division, while *ins:*YFP fluorescence in the green channel was quantified. For experiments requiring cell counting in the two halves, the divider was overlaid back onto the 3D image stack, and the cells were manually counted through the stack.

### Two-photon laser ablation of the zebrafish primary islet

Embryos from *Tg(ins:BB1.0L; cryaa:RFP); Tg(ins:Cre-ER* ^*T2*^ *; cryaa:CFP)* were treated with 0.003% (200 µM) 1-phenyl 2-thiourea (PTU) at 10 hpf to inhibit pigmentation. Embryos were treated with 1 µM 4-OHT + 0.003% PTU at 24 hpf, as described before to induce *Beta-bow* recombination in dorsal bud-derived beta-cells (DBCs). At 2 dpf islets were subjected to two-photon laser-induced injury using Leica SP5 MP equipped with an objective (25×/0.95 N.A.). For this, the embryos were anaesthetized and mounted in 1.5% low-melting agarose. Designated area (2–5 cells) of the on-focus *z*-plane within the tissue was exposed to pulsed two-photon laser at the output power of 2.0 W (*λ* = 800 nm) for 2 min. To avoid depletion of DBCs, the designated area, and thus photoablation, was restricted to the unlabeled beta-cells, detected by their lack of recombination label (CFP + YFP). The embryos were carefully removed from agarose, washed in E3 and placed in fresh dish with E3 to develop till 3.5 dpf, when they were fixed for further analysis. Control animals were mounted and imaged with the two-photon laser turned off to avoid islet injury.

### Pharmacological treatments

The following compounds were used: Harmine (Tocris, 5075), LY411575 (Sigma, SML0506), and EP (Sigma, E47808). Treatment with harmine was performed from 3 to 5 dpf with fresh solution added once at 4.5 dpf. Treatment with LY411575 and EP was from 15 to 18 dpf. Fresh solution was added for 12 h (overnight), followed by washing and returning to regular fish water with feeding during the day. DMSO was used as a control.

### Genetic ablation of beta-cells

For beta-cell ablation, fish were incubated with 10 mM Mtz (Sigma, M1547) dissolved in aquarium water, and maintained for 24 h in the dark before they were rinsed and returned to recirculating aquarium water.

### Immunofluorescence and image acquisition

Whole-mount immunofluorescence was performed on pancreas collected as described above. The collected samples were permeabilized in 1% PBT (Triton-X-100) and blocked in 4% PBTB (BSA). Primary and secondary antibody stainings were performed overnight at 4 °C. Primary antibodies used in this study were anti-insulin (guinea pig, Dako A0564) at 1:200, anti-mCherry (rabbit, Takara 632496) at 1:200, and anti-EGFP (chicken, Abcam ab13970) at 1:250, anti-Nkx6.1 at 1:20 (mouse, clone F55A10), anti-glucagon (mouse, Sigma) at 1:200 and anti-urocortin3 (rabbit, Phoenix Peptide H-019-29 Urocortin III) at 1:300. This polyclonal antibody was chosen because the peptide that was used for its generation contains a stretch containing approximately 30 identical aminoacids between zebrafish and mouse. Anti-urocortin3 was first validated using cryosections of adult islets stained with the insulin antibody and a GFP reporter marking beta-cells, as well as in whole mounts using 5 dpf larvae. A time course analysis at 24, 36, 48, 60, and 72 hpf showed that it consistently labeled beta-cells at 72 hpf but not earlier, in agreement with the timing of expression of *ucn3* at 72 hpf, determined using in situ hybridization. Secondary antibodies used in this study were Alexa Fluor 405 and Alexa Fluor 488 anti-guinea pig (1:200), Alexa Fluor 568 anti-rabbit (1:200) and Alexa Fluor 488 anti-chicken (1:200), and Alexa Fluor 647 anti-mouse (1:200). Samples were mounted in Fluoromount-G or Vectashield and imaged using a Zeiss LSM 780. ImageJ was used to add scale bars and PowerPoint was used for adding arrows and labels.

### Cell counting

Total number of beta-cells in the islets were counted using Imaris (Bitplane). For counting beta-cells in *Tg(ins:FUCCI-G1);Tg(ins:FUCCI-S/G2/M)*, the islet was divided into two equal halves by calculating the distance between the anterior most to the posterior most cells. The “spots” function of Imaris, with appropriate thresholding, was used to count all the red cells in stacks spanning the entire islet. All the proliferating cells (*green* only) were counted manually. This approach enabled us to quantify the percentage of proliferating beta-cells in the whole islet. For the 27 dpf samples, we re-analyzed confocal stacks acquired during a previous study^32^. In all samples, including 23 and 35 dpf, the fish were fed two times with *Artemia* (brine shrimp) during the day and the samples were collected ~10 h after the first feeding, which is important to trigger beta-cell proliferation^32^. The same feeding regimen was applied during the experiments in which proliferation was studies using PCNA.

### EdU labeling

To label proliferating cells in larval zebrafish, the larvae were placed in E3 media with 2.5 mM EdU from 3 to 5 dpf. The larvae were fixed in 4% paraformaldehyde + 1% Triton-X overnight. After 3 × 10 min PBS washes, EdU detection was performed according to the kit protocol Click-iT EdU Alexa Fluor 647 Imaging Kit (C10340 Fisher Scientific). Following the EdU labeling protocol, the samples were stained with an anti-EGFP antibody to recover the GFP fluorescence loss resulting from the Click-iT detection reaction.

### PCNA labeling and quantification

To label proliferating cells in 30 dpf pancreatic islets from *Tg(ins:Cre-ER* ^*T2*^ *);Tg(ins:mCherry-Stop-H2B-EGFP)* animals, PCNA staining was performed on fixed islets. Antigen retrieval was performed by transferring the islets to 10 µM acetic acid and heating the samples to 85 °C for 10 min in a table-top heat block. After 3 × 10 min PBS washes, PCNA antibody staining was performed according to the standard protocol. Anti-PCNA (mouse, Abcam ab29) was used at 1:300 dilution, along with anti-EGFP antibody to recover the loss of GFP fluorescence from the detection protocol and anti-insulin antibody to label all beta-cells. Confocal stacks spanning the entire islet were used for quantification. The PCNA-positive beta-cells were counted manually, while the counting of the EGFP-positive and RFP-positive cells were performed using the Imaris Imaging Software (Bitplane).

### Whole-mount in situ hybridization

*Probe preparation. ucn3* ^37^ cDNA was PCR amplified using primers with an Sp6-promoter sequence and a T7-promoter sequence, respectively, tagged onto the 5′-end (ucn3l-fw: 5′- ATTTAGGTGACACTATAGtctctctcgcctccgttctccaa-3′; ucn3l-rv: 5′-TAATACGACTCACTATAGggagatcaaattggtgacacgaacaca-3′). DIG RNA labeling mix (Roche) was used to generate DIG-labeled *ucn3l* probes by in vitro transcription with T7 RNA polymerase (Fermentas). RNA probes were purified using Micro Bio-Spin30 Columns, Tris, RNase-free (Bio-Rad). For the insulin probe, cDNA from 24 hpf embryos was PCR amplified using the following primers: insa-fw: 5′-atggcagtgtggcttcaggc-3′; insa-rv: 5′-gaattctcagttacagtagt-3′. PCR products of the correct size were purified from agarose gels using the QIAquick Gel Extraction Kit (QIAGEN), and subcloned using the Dual Promoter TA Cloning kit (Invitrogen). Plasmids were linearized using the appropriate enzyme and fluorescein-labeled probes were generated by in vitro transcription using T7 RNA polymerase (Fermentas).

### In situ hybridization

Embryos were dechorionated manually. Embryos and larvae were fixed in RNAse-free 4% PFA at the desired developmental stages for 24 h at 4 °C, dehydrated using a methanol gradient and stored at −20 °C for at least 24 h. After rehydration, larval pigments were removed by incubating the larvae in 3% H_2_O_2_; 1%KOH; ddH_2_O for 10–15 min, and washing several times with 1× PBS. Samples were then incubated in proteinase K (24 hpf embryos:10 min; 3 and 5 dpf larvae: 30 min), which was washed out by 1× DEPC-PBST. After re-fixing the samples in 4% PFA for 20 min at RT, PFA was washed out with 1× DEPC-PBST. Samples were then incubated in hybridization (Hybe) buffer for 2 h at 65 °C. Shortly before applying the probes (1 ng/μl), each probe was denatured in Hybe for 10 min at 75 °C. Samples were then incubated simultaneously with DIG-labeled *ucn3l*—and fluorescein-labeled insulin probes in Hybe solution O.N. at 65 °C. Samples were then washed at 65 °C in: (3× SSC/50% formamide/0.1% Tween-20); (2× SSC/50% formamide/0.1% Tween-20); (2× SSC/0.1% Tween-20; 0.2× SSC/0.1% Tween-20), followed by washes with 1× PBST and subsequent washes with maleic buffer at RT. Afterwards, samples were incubated in 2% blocking buffer (Roche) for 2 h at RT. To detect the DIG-labeled RNA probes, samples were incubated in anti-DIG-AP-fab fragments (Roche) diluted 1:5000 in blocking buffer overnight at 4 °C. Samples were rinsed with maleic buffer, 1× PBST and NTMT. For staining the substrates, NBT and BCIP were added to the NTMT buffer. Signal development was monitored every 15 min under a stereomicroscope and stopped with 1× PBST washes. To detect the insulin signal, samples were then incubated in anti-Fluorescein AP (Roche) diluted 1:2000 in blocking buffer O/N at 4 degrees. For fluorescein signal detection, samples were incubated in NTMT staining buffer with the substrates INT and BCIP. Signal development was monitored under a stereomicroscope and stopped with washes in 1× PBST.

### GCAMP6s image acquisition and analysis

To monitor the changes in glucose-stimulated calcium influx during development, GCAMP6s measurements were performed on isolated islets from *Tg(ins:gCaMP6s; cryaa:mCherry); Tg(ins:Renilla-mKO2; cryaa:CFP)* double-transgenic animals at 4, 25, 35, and 45 dpf. Freshly dissected islets from killed fish were washed with HBSS containing Ca2+/Mg2+ (Life Technologies, 14175095) twice and embedded in fibrin gels (3:1 ratio of 10 mg/ml Bovine fibrinogen, 50 U/ml Bovine thrombin; Sigma Aldrich). Upon polymerization, islets were immersed in HBSS containing 5 mM glucose, visually oriented along A/P axis and imaged using live confocal microscopy (LSM 780 FLIM inverse) to establish the baseline. The islets were then stimulated with 10 and 20 mM d-glucose (final concentration; Sigma, G8270), and finally with 30 mM KCl (final concentration; Sigma, P9451) without changing the solution. For quantifying fluorescence dynamics, ROIs were manually selected around individual beta-cells in Fiji. Baseline intensity (*F*_0_) was calculated as the mean of fluorescence intensity at 5 mM glucose. Baseline intensity was subtracted from stimulus-induced calcium intensity changes at 10, 20 mM glucose, and 30 mM KCl (*F*–*F*_0_), and normalized to the maximum intensity (*F*_max_−*F*_0_). Only images that captured the entire islet were used for quantification. In all GCAMP experiments, cells that failed to show fluorescence enhancement upon KCl stimulation were not used for analysis and quantification.

To monitor glucose-stimulated calcium influx in H2BmChery-positive and -negative cells, GCAMP6s measurements were performed on isolated islets from *Tg(ins:gCaMP6s; cryaa:mCherry); Tg(ins:CFP-NTR)* double*-*transgenic animals injected with H2B-mCherry mRNA at the one-cell stage. Freshly dissected islets from killed animals were washed with HBSS containing Ca2+/Mg2+ twice and embedded in fibrin gels. Upon polymerization, islets were immersed in HBSS containing 5 mM glucose and visually oriented along the A/P axis. Z-scans covering the entire volume of the islets were performed in the mCherry and the CFP channels in order to quantify H2B-mCherry fluorescence expression in beta-cells. Live confocal microscopy was then carried out using the GFP, mCherry, and CFP channels to visualize the GCaMP signal and the beta-cells, respectively. The islets were then stimulated with 7.5 mM d-glucose (final concentration) and finally with 30 mM KCl (final concentration) without changing the solution. Z-scans were used to mark individual cell nuclei and to manually quantify the mean mCherry fluorescence and standard deviation per unit area using ImageJ. A cell was considered as H2B-mCherry-negative if the mean was similar to background. The cells belonging to the H2B-mCherry-positive and -negative categories were cross-referenced manually in the GCaMP6s videos, and quantified for changes in stimulus-induced calcium intensity as above.

### H2B-mCherry mRNA injection

To label quiescent cells, the H2B-mCherry mRNA was injected in the one-cell stage embryos using standard protocols. In brief, pCS2+-H2B-mCherry plasmid was maxi-prepped, digested with KpnI and in vitro transcribed using mMESSAGE mMACHINE SP6 Transcription Kit (Ambion, AM1340) to generate mRNA. About 500 pg of H2B-mCherry mRNA was injected in each embryo. No antibody staining was used to detect protein expression.

### Statistical analysis

Statistical analysis was performed using R. No animals were excluded from analysis. Blinding was not performed during analysis. Analysis of normal distribution was performed. To compare clonal properties (single cells vs. multicellular clone) between two time points, Fisher’s exact test for count data (Fisher’s.test (*x* = 2×2 matrix, alternative = “two.sided”)) was performed. To compare the posterior/anterior ratio of glucose responsiveness between 25, 35, and 45 dpf, a Kruskal–Wallis test (Kruskal.test (*x* = numeric vector, *g* = factor vector)) was first performed, followed by Wilcox test (Wilcox.test (*x* = numeric vector, *y* = numeric vector)) in pair-wise fashion. For all comparisons related to differences along the A/P axis, a paired two-tailed *t*-test with unequal variance (*t*-test (*x* = data frame, alternative = “two.sided”, paired = TRUE, var.equal = FALSE)) was used to calculate *p*-values. All other comparisons utilized unpaired two-tailed *t*-test with unequal variance (*t*-test(*x* = dataframe, alternative = “two.sided”, paired = FALSE, var.equal = FALSE)). All results are presented as mean ± standard error of the mean unless otherwise indicated. A *p*-value of <0.05 was considered statistically significant. For plotting, Tukey style boxplots showing the median along with the 25th percentile (lower quartile - Q1) to 75th percentile (upper quartile - Q3) range were used. For all boxplots, whiskers extend to 1.5 times the interquartile range (IQR = Q3 − Q1)

### Data availability

All relevant data and reagents are available from the authors upon request.

## Electronic supplementary material

Supplementary Information

Supplementary Movie 1

Supplementary Movie 2

Supplementary Movie 3

Supplementary Movie 4

Supplementary Movie 5
