# Supplementary material for: Different developmental histories of beta-cells generate functional and proliferative heterogeneity during islet growth
Source: Nat Commun. 2017 Sep 22;8:664. doi: 10.1038/s41467-017-00461-3 (PMC5610262; doi:10.1038/s41467-017-00461-3)
Supplement: Supplementary file 1 — Supplementary Information [file 41467_2017_461_MOESM1_ESM.pdf]

Supplemental Figures:

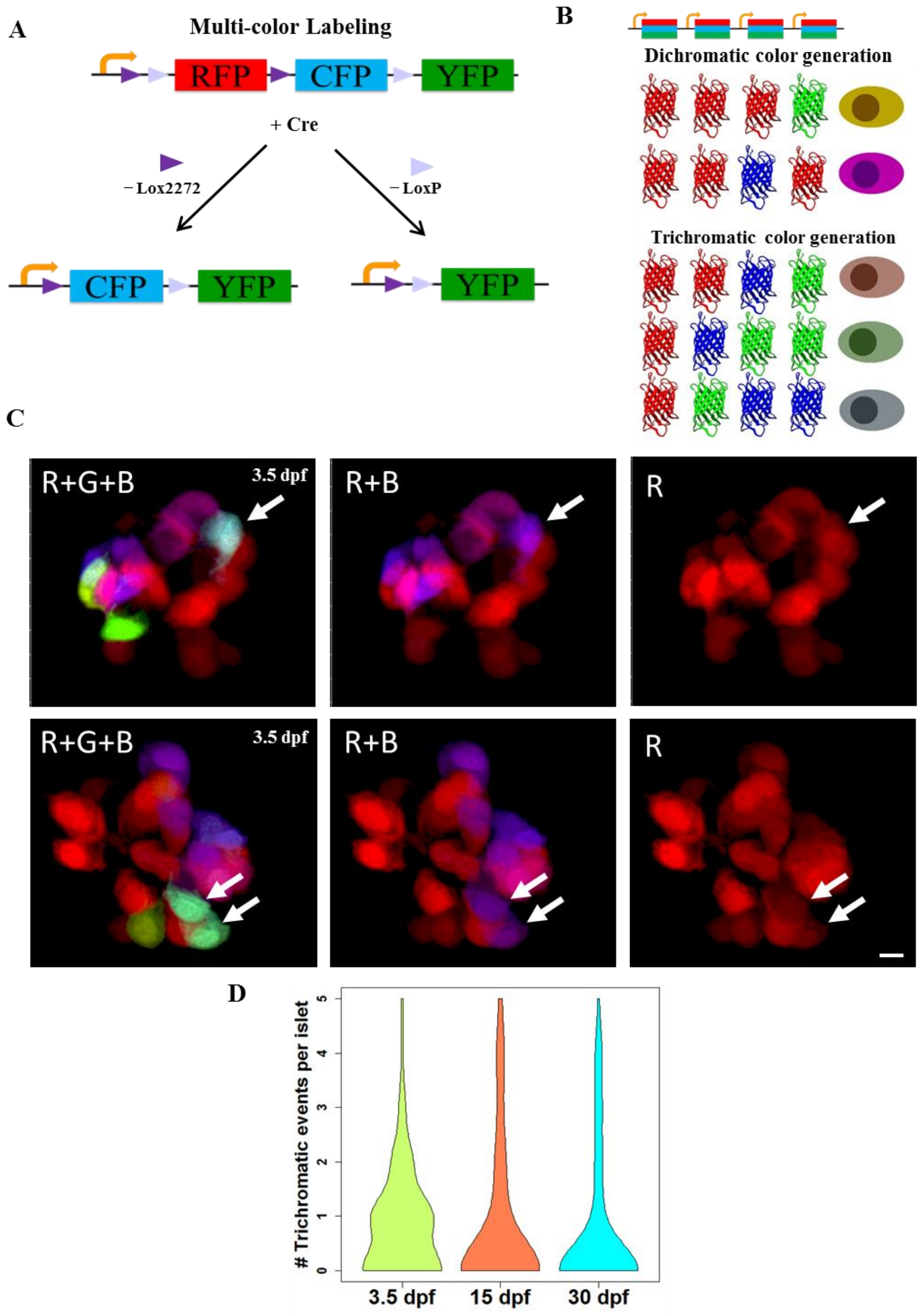

Supplementary Figure 1: The *Beta-bow* system for unique labeling of individual beta-cells.

(A-B) Schematic of multicolor labeling using *beta-bow*. **(A)** Induction of Cre recombinase (driven by the *insulin* (*ins*) promoter and activated by 4-OHT treatment) excises the DNA flanked by either Lox2272 (►) or LoxP (►), thereby switching RFP expression to CFP or YFP, respectively within the beta-cells. **(B)** Presence of several floxed cassettes in each cell leads to multiple discrete recombination events, which result in the combinatorial expression of fluorescent proteins and unique labeling of beta-cells in different colors. The expression of either green or blue fluorescence together with the red color in beta-cells can arise after a

single-recombination event. In contrast, the induction of trichromatic cells, which express the green, blue and red colors together, requires at least two independent recombination events in the same cell.

(C) Confocal projections of *beta-bow* islets showing trichromatic events, either single beta-cells (top panels) or pairs of cells, which consist of two cells with the same color combination (bottom panels). *Beta-bow* recombination was induced using a treatment with 4-OHT at 24 hours post-fertilization (hpf). The samples were analyzed at 3.5 days post-fertilization (dpf). In 105 recombined islets, there were 85 trichromatic events, of which eighty were singlets and five were pairs of cells, each of the pairs consisting of two cells with the same color combination.

(D) Violin plots showing the probability density (distribution) of the numbers of trichromatic events (single cells or multicellular clones) per recombined islet at 3.5, 15 and 30 dpf ($n>50$ islets per stage from three independent experiments). Recombination was induced by treatment with 4-OHT from 24 to 30 hpf. The mean frequency of trichromatic events does not appear to change significantly between time points. The mean values for trichromatic events per islet were 0.8 (3.5 dpf), 0.87 (15 dpf) and 0.77 (30 dpf). Scale bars, 5 µm.

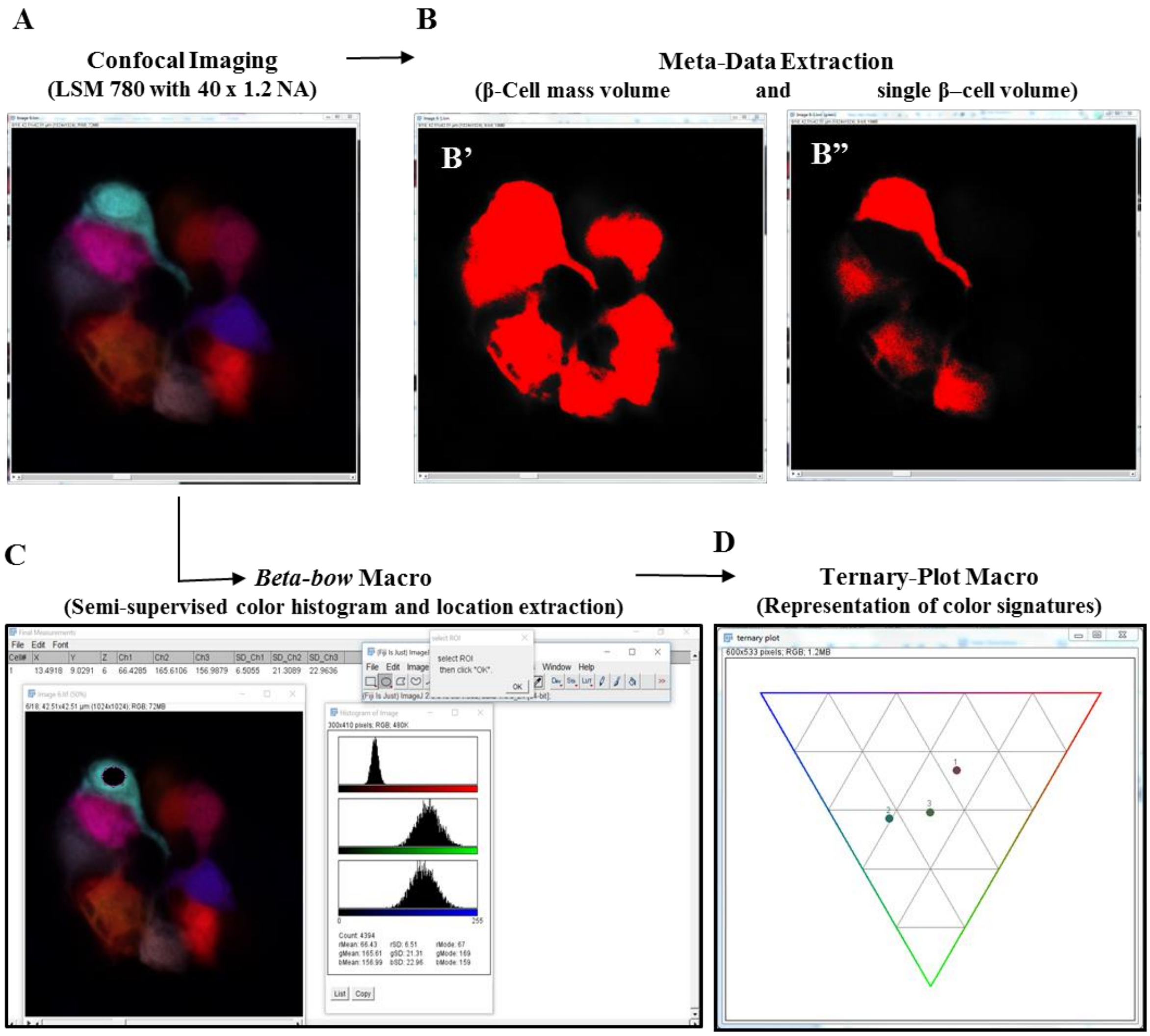

Supplementary Figure 2: Setup for image analysis and data processing using the *Beta-bow* system.
(A-D) Schematic of data extraction and analysis using the *Beta-bow* system.
(A) *Beta-bow* samples were imaged using a Zeiss LSM 780 confocal microscope with a 40 x 1.2 NA water-objective. The confocal images were analyzed using ImageJ macros developed in-house.
(B) The meta-data for volumetric measurements were obtained by thresholding the images.
(B') Cells containing red, blue and green colors were used for extracting the volume of the total beta-cell mass in the primary islet. **(B")** The color channels were separated in ImageJ, and the green or blue colors were used for measuring the single-cell volume of beta-cells.
(C) For determining the color signatures of beta-cells, the nuclei of the multicolor beta-cells were manually outlined and the information was transferred to a macro for extracting and saving the regional color histogram. Only those cells that exhibit trichromatic color signatures were used for clonal analysis.
(D) Visual representations of color signatures were made possible by plotting normalized values on a ternary plot.

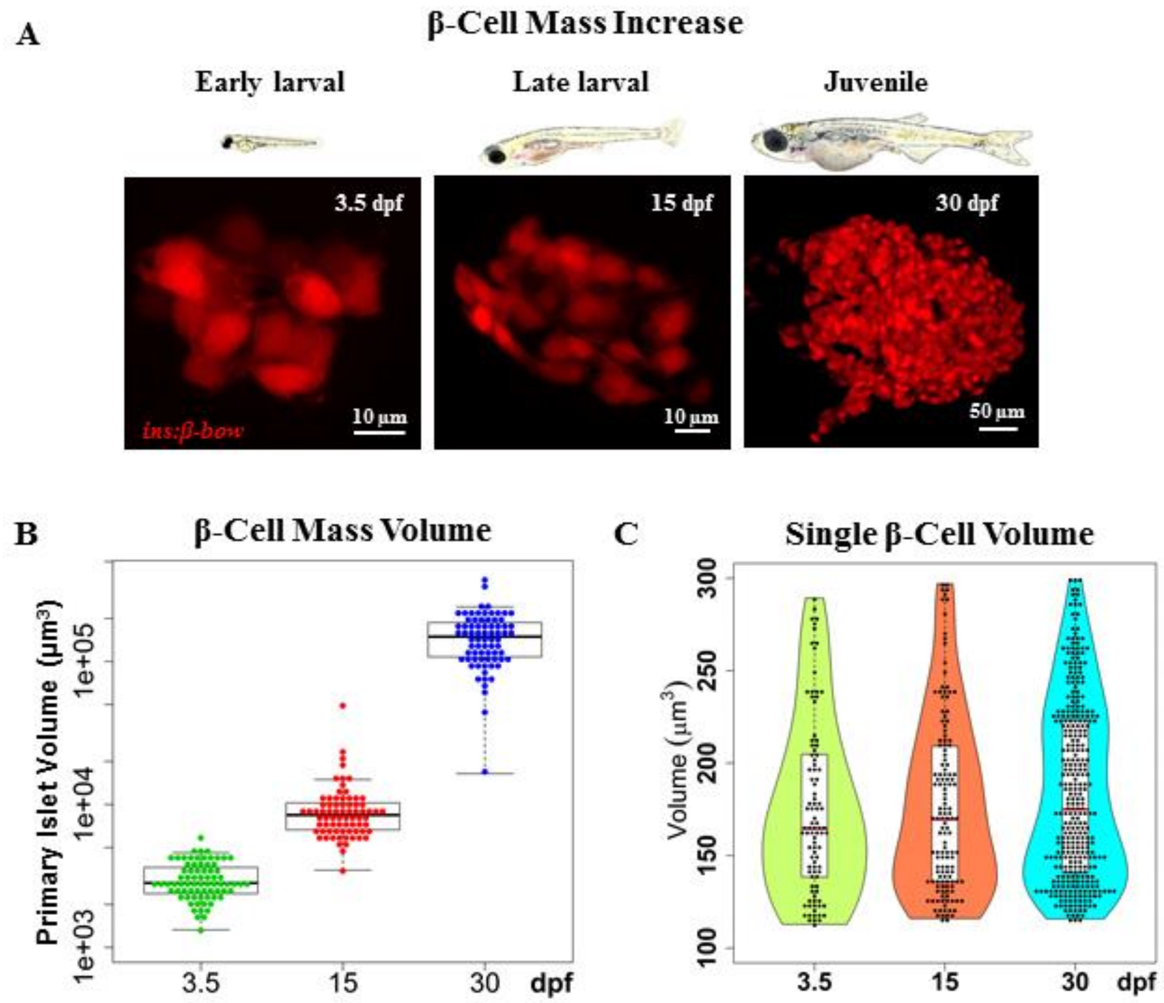

Supplementary Figure 3: The increase in beta-cell mass during zebrafish development occurs in the absence of beta-cell hypertrophy.

(A) Top – cartoons (to scale) depicting zebrafish at three stages of development: 3.5, 15 and 30 dpf. Bottom - representative maximum intensity projections of the primary islet from *Tg(ins:BB1.0L)* at each stage.

(B) Quantification of the total beta-cell mass in the primary islet during zebrafish development. The plot shows tukey style boxplot overlaid with the data points. This analysis shows a 3-fold increase from 3.5 to 15 dpf, and a 16.7-fold increase from 15 to 30 dpf; resulting in a total increase of approximately 50-fold over the observed period.

(C) Quantification of single beta-cell volume during the three stages of zebrafish development. Violin plots show the probability density (distribution) of cell sizes at each stage. The median and mean single-cell volume does not statistically differ among the three stages. To mark beta-cells in multiple colors in order to measure single beta-cell size, *beta-bow* recombination was induced using 4-OHT-treatment at 24 hpf. Scale bars, 10 µm.

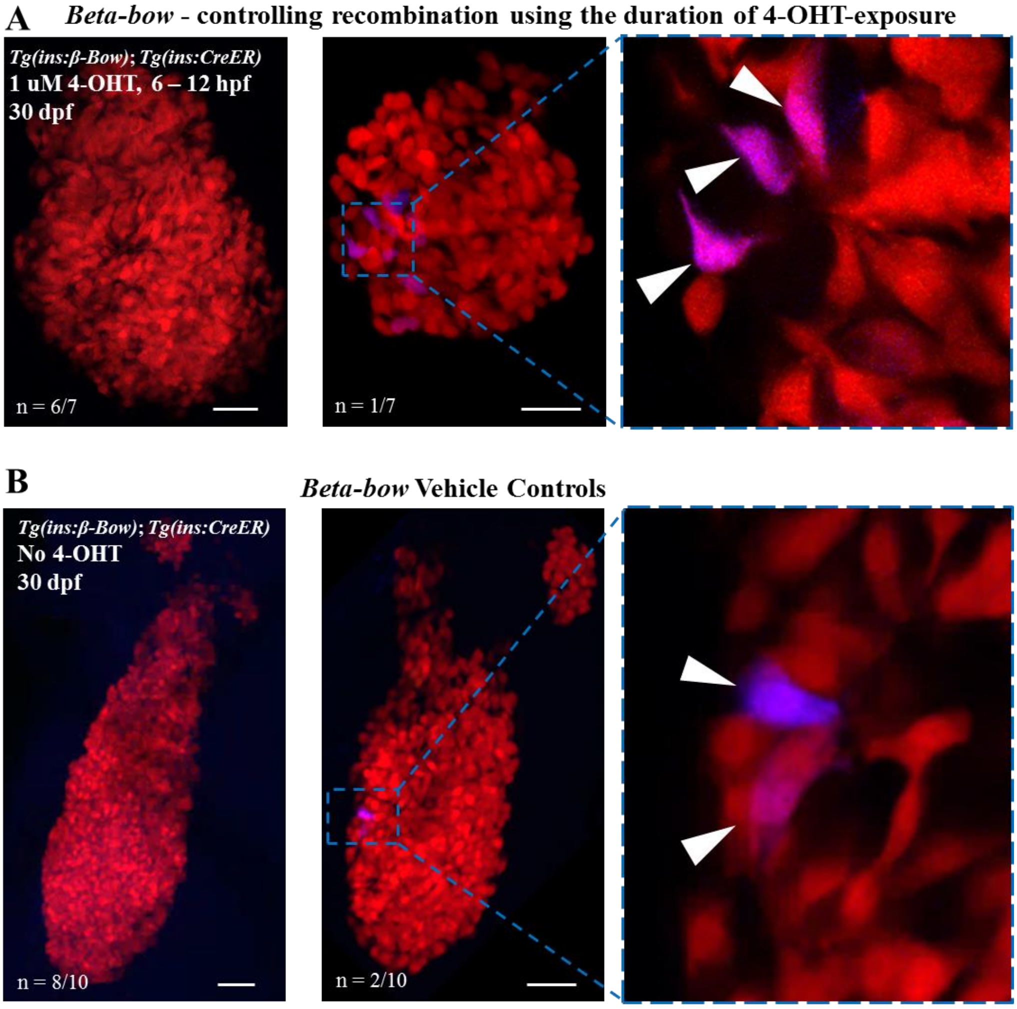

Supplementary Figure 4: Controls for the timing of recombination using the duration of 4-OHT-exposure; analysis of background-recombination in the *Beta-bow* system at 30 dpf.

(A) Maximum intensity projection of the islets from 30 dpf *Tg(ins:beta-bow)*; *Tg(ins:CreER^{T2})* animals. Embryos were incubated with 1 uM 4-OHT from 6 to 12 hpf. The 4-OHT was washed away at 12 hpf, two hours before the onset of *insulin* expression in the dorsal-bud-derived beta-cells. 6 out of 7 islets (left) showed no recombination, while 1 out of 7 islets (right) displayed six cells (arrowhead) exhibiting background recombination. All cells with background recombination were dichromatic. No trichromatic cells were observed.

(B) Maximum intensity projection of 30 dpf islets from *Tg(ins:beta-bow)*; *Tg(ins:CreER^{T2})* animals showing low leaky recombination in the absence of 4-OHT-incubation, as assessed by the expression of CFP or YFP. 8 out of 10 islets (left) showed no recombination, while 2 out of 10 islets (right) displayed two cells each (arrowhead) exhibiting background recombination. All cells exhibiting background recombination were dichromatic. No trichromatic cells were observed. Dichromatic cells can form as a result of a single-recombination event in *Tg(ins:beta-bow)*. The probability of a single-recombination event was estimated to be ≈ 0.0005 (see materials and methods). Trichromatic cells require at least two recombination events to form. The probability of two recombination events without 4-OHT-induction was estimated to be 2.5E-7 (see materials and methods). Scale bars, 50 µm.

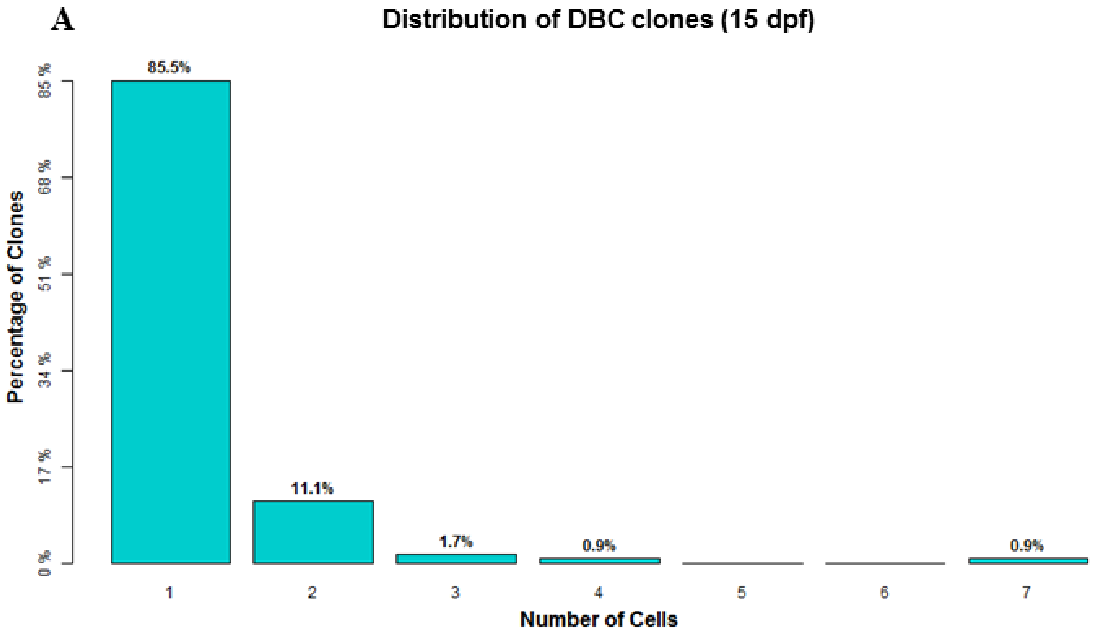

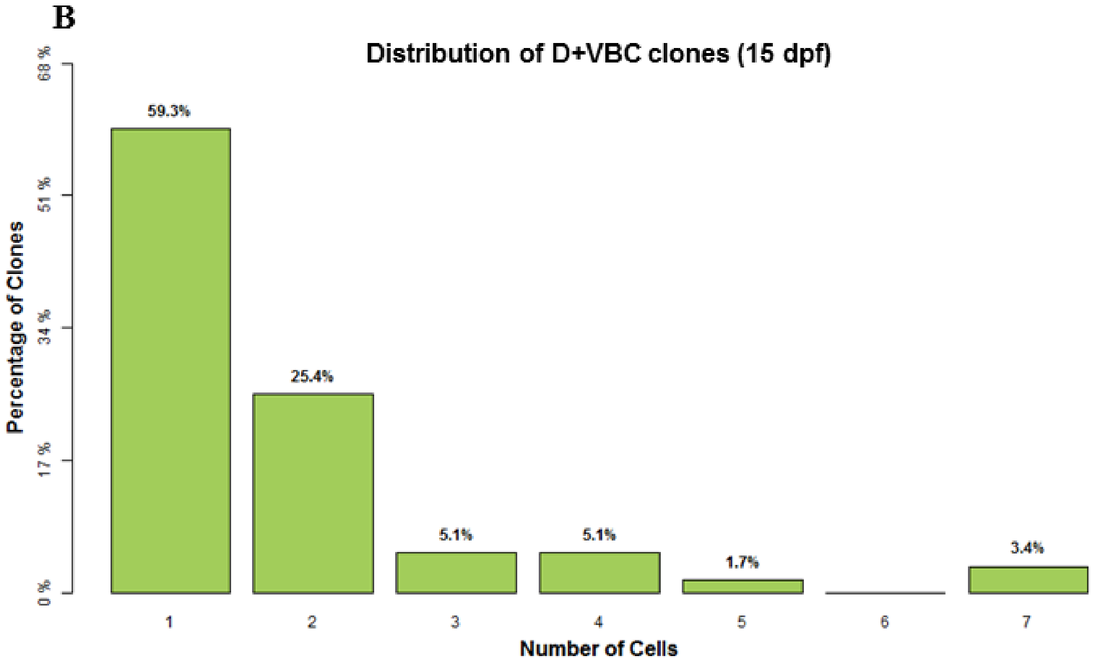

Supplementary Figure 5: Embryonic beta-cells exhibit diverse contributions to the growth of the islet at 15 dpf.

(A) Clonal distribution analysis for the dorsal bud-derived beta-cells (DBCs) at 15 dpf. DBCs were labeled at 24 hpf by incubating *beta-bow* animals with 4-OHT for 6h. Samples were collected at 15 dpf. The trichromatic cells were used for clonal analysis. The X-axis shows the clone size. The Y-axis indicates the percentage.

(B) Clonal distribution analysis for the combined population of dorsal and ventral bud-derived beta-cells (D+VBCs) at 15 dpf. D+VBCs were labeled at 48 hpf by 4-OHT incubation for 3h. Samples were collected at 15 dpf. The trichromatic cells were used for clonal analysis. The X-axis shows the clone size. The Y-axis indicates the percentage.

A

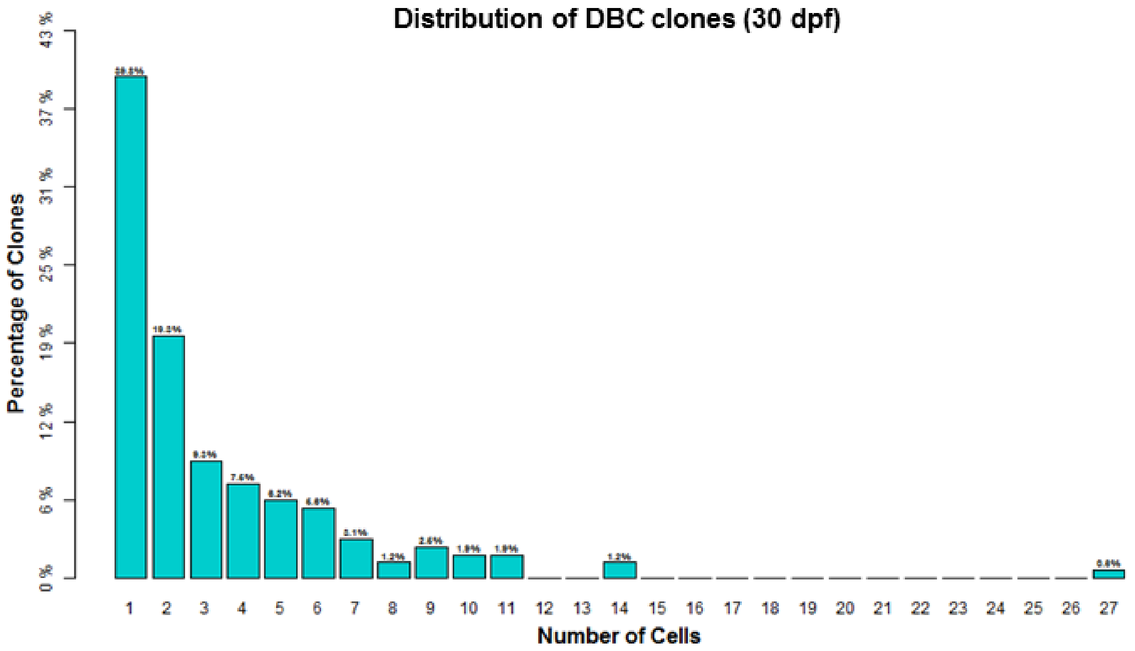

B

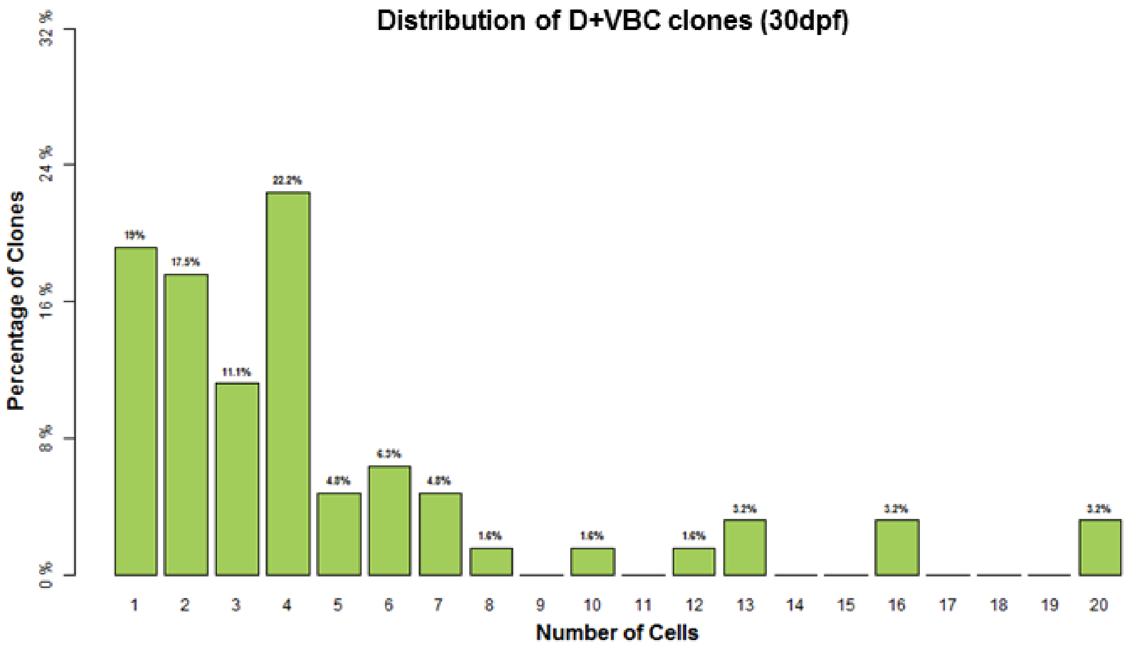

Supplementary Figure 6: Embryonic beta-cells exhibit diverse contributions to the growth of the islet at 30 dpf.
(A) Clonal distribution analysis for the dorsal bud-derived beta-cells (DBCs) at 30 dpf. DBCs were labeled at 24 hpf by incubating *beta-bow* animals with 4-OHT for 6h. Samples were collected at 30 dpf. The trichromatic cells were used for clonal analysis. The X-axis shows the clone size. The Y-axis indicates the percentage.
(B) Clonal distribution analysis for the combined population of dorsal and ventral bud-derived beta-cells (D+VBCs) at 30 dpf. D+VBCs were labeled at 48 hpf by 4-OHT incubation for 3h. Samples were collected at 30 dpf. The trichromatic cells were used for clonal analysis. The X-axis shows the clone size. The Y-axis indicates the percentage.

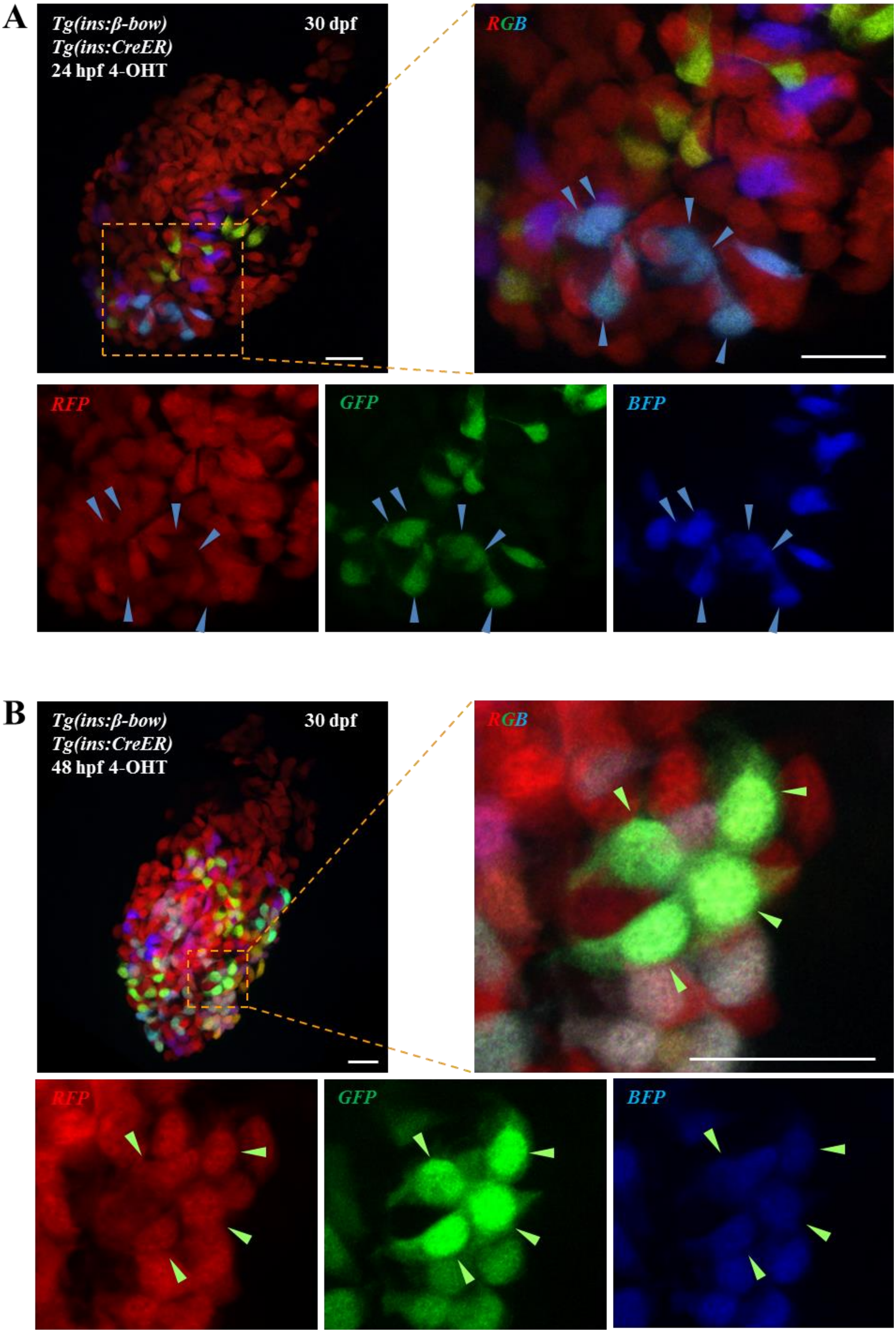
A
Tg(ins:β-bow)
Tg(ins:CreER)
24 hpf 4-OHT
30 dpf
RGB
RFP
GFP
BFP
B
Tg(ins:β-bow)
Tg(ins:CreER)
48 hpf 4-OHT
30 dpf
RGB
RFP
GFP
BFP

Supplementary Figure 7: Representative trichromatic multicellular clones at 30 dpf shown in Figs 1C and 1E.

(A) A primary islet from *beta-bow* animals at 30 dpf with the DBCs labeled in multiple colors. The inset shows an enlarged view of the area outlined with a dashed line. The color channels are shown separately in the bottom panels. Arrowheads - trichromatic beta-cells forming a multicellular clone.

(B) A primary islet from *beta-bow* animals at 30 dpf with D+VBCs labeled in multiple colors. The inset shows an enlarged view of the area outlined with a dashed line. The color channels are shown separately in the bottom panels. Arrowheads - trichromatic beta-cells forming a multicellular clone. Scale bars, 20 µm.

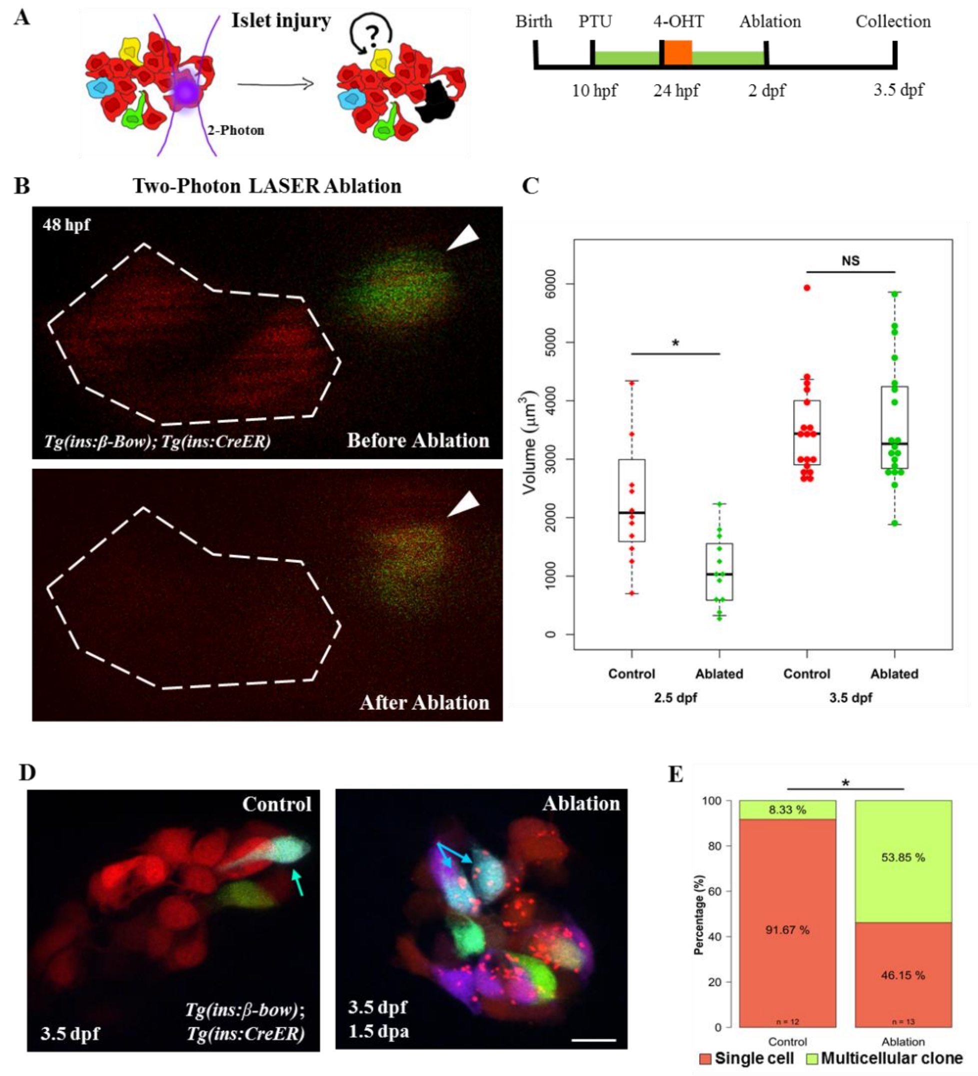

Supplementary Figure 8: Injury based reversal of DBCs quiescence.

(A) Cartoon (left) and a schematic (right) for the two-photon laser-injury model. The model tests if partial islet-ablation is capable of inducing a proliferative response among the quiescent dorsal bud-derived beta-cells (DBCs).

(B) Snapshots from in-vivo imaging of 48 hpf *beta-bow* animals undergoing two-photon laser-ablation. DBCs were labeled in multiple colors at 24 hpf by 4-OHT-incubation. The dotted line indicates the target area, composed of un-recombined cells (red). Arrowheads point to recombined cells, located outside of the target area. These cells were not ablated.

(C) Quantification of the total volume of the beta-cells in the primary islet following laser-ablation. The plot shows tukey style boxplot overlaid with the data points. At 12 hours post-ablation (2.5 dpf), the ablated islets exhibited smaller volumes compared to the control islets (unpaired t-test,* $p \leq 0.05$). At 36 hours post-ablation (3.5 dpf), the volume of beta-cells in the ablated samples was similar to controls.

(D) Maximum intensity projections of control and ablated primary islets. Arrows indicate trichromatic cells. Note the presence of red puncta in the beta-cells from the laser-ablated islets, which might indicate aggregation of misfolded proteins due to induction of cellular stress after the injury.

(E) Quantification of the proportion of trichromatic beta-cells that remain as single cells or form multicellular clones in the control and laser-ablated islets. Multicellular clones denote at least two cells per clone. Approximately one-half of DBCs underwent cell divisions in response to injury, a statistically significant increase in proliferation compared to controls (Fisher's exact test,* $p \leq 0.05$). Scale bars, 10 µm

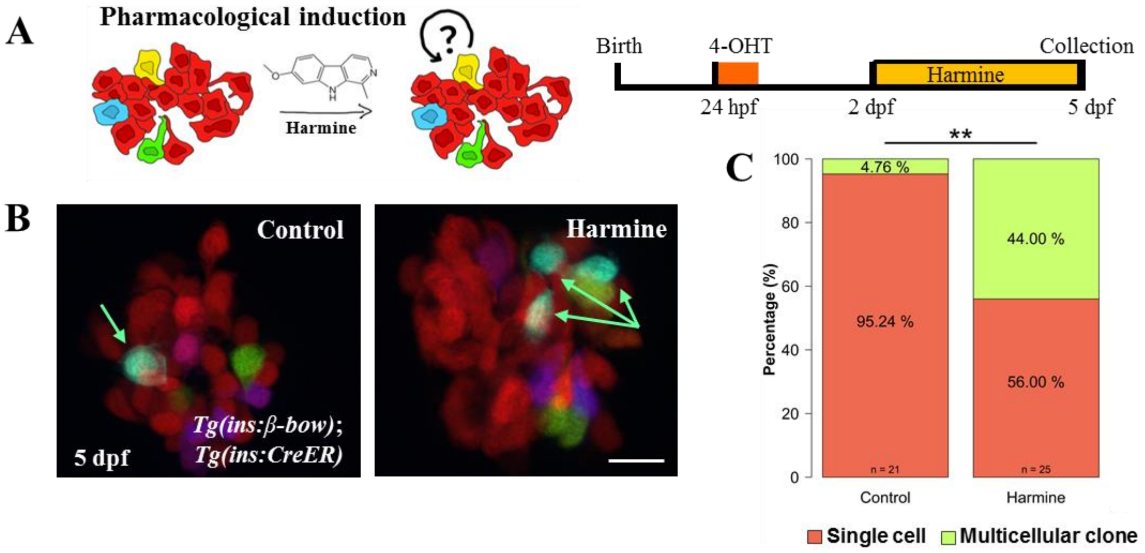

Supplementary Figure 9: Small molecule based reversal of DBCs quiescence using harmine.

(A) Cartoon (left) and schematic (right) for the pharmacological stimulation of proliferation. Dorsal bud-derived beta-cells (DBCs) were labeled by incubating *beta-bow* animals with 4-OHT at 24 hpf. Subsequently, the animals were incubated from 2–5 dpf with Harmine or DMSO.

(B) Maximum intensity projections of the primary islets from control and harmine-treated *beta-bow* animals. Arrows indicate trichromatic cells.

(C) Quantification of the proportion of trichromatic beta-cells that remain as single cells or form multicellular clones (≥ 2 cells/clone) in the control and laser-ablated islets. Harmine-treatment increased the percentage of multicellular clones compared to DMSO (Fisher's exact test, ** $p \leq 0.01$). Scale bars, 10 µm.

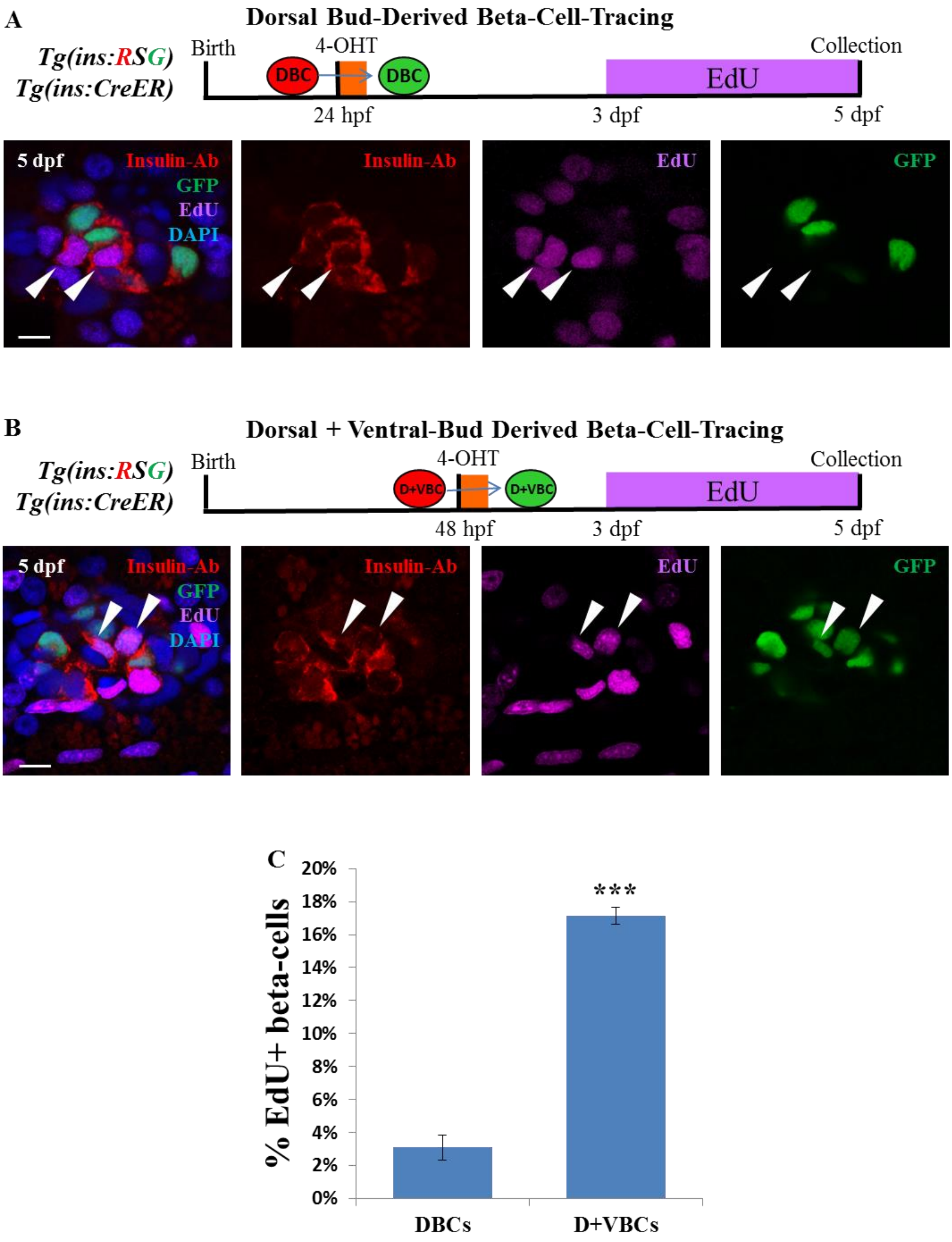

Supplementary Figure 10: Analysis of beta-cell proliferation in DBCs and the combined D+VBCs population during an equivalent developmental window.

(A) Top - schematic showing the rationale for studying the proliferation of DBCs. *Tg(ins:mCherry-Stop-H2B-EGFP); Tg(ins:CreER^{T2})* animals were incubated with 1 uM 4-OHT from 24 to 30 hpf in order to label specifically the DBCs with nuclear GFP fluorescence. The larvae were placed in 2.5 mM EdU from 3 to 5 dpf to label all proliferating beta-cells.

Bottom - a single confocal section showing GFP-positive DBCs at 5 dpf. The DBCs (green) are EdU-negative. In contrast, the adjacent GFP-negative beta-cells are EdU-positive. Note that the red (mCherry)-fluorescence from *Tg(ins:mCherry-Stop-H2B-EGFP)*was lost due to the Click-iT reaction used to reveal EdU-incorporation. The GFP-signal was recovered using an anti-GFP antibody. All beta-cells were marked using an anti-insulin antibody.
(B) Top - schematic showing the rationale for studying the proliferation of beta-cells in the combined D+VBCs population. *Tg(ins:mCherry-Stop-H2B-EGFP); Tg(ins:CreER^{T2})* embryos were incubated with 2 uM 4-OHT at 48 hpf in order to label the D+VBCs with nuclear GFP fluorescence. Larvae were placed in 2.5 mM EdU from 3 – 5 dpf to label all proliferating beta-cells.
Bottom - a single confocal section showing GFP-positive beta-cells from the combined D+VBC population at 5 dpf. Arrowheads indicate EdU and GFP-double-positive beta-cells. Note that the red (mCherry)-fluorescence from *Tg(ins:mCherry-Stop-H2B-EGFP)* was lost due to the Click-it reaction used to reveal EdU-incorporation. The GFP-signal was recovered using an anti-GFP antibody. All beta-cells were marked using an anti-insulin antibody.
(C) Quantification of the percentage of EdU and GFP-double-positive beta-cells from the experiments in A and B. The D+VBCs population contains a higher proportion of proliferating beta-cells as compared to the DBC population alone (unpaired t-test, *** $p \leq 0.001$). $n \geq 5$ islets at each stage with ≥10 GFP-positive cells per islet. Scale bars, 10 µm.

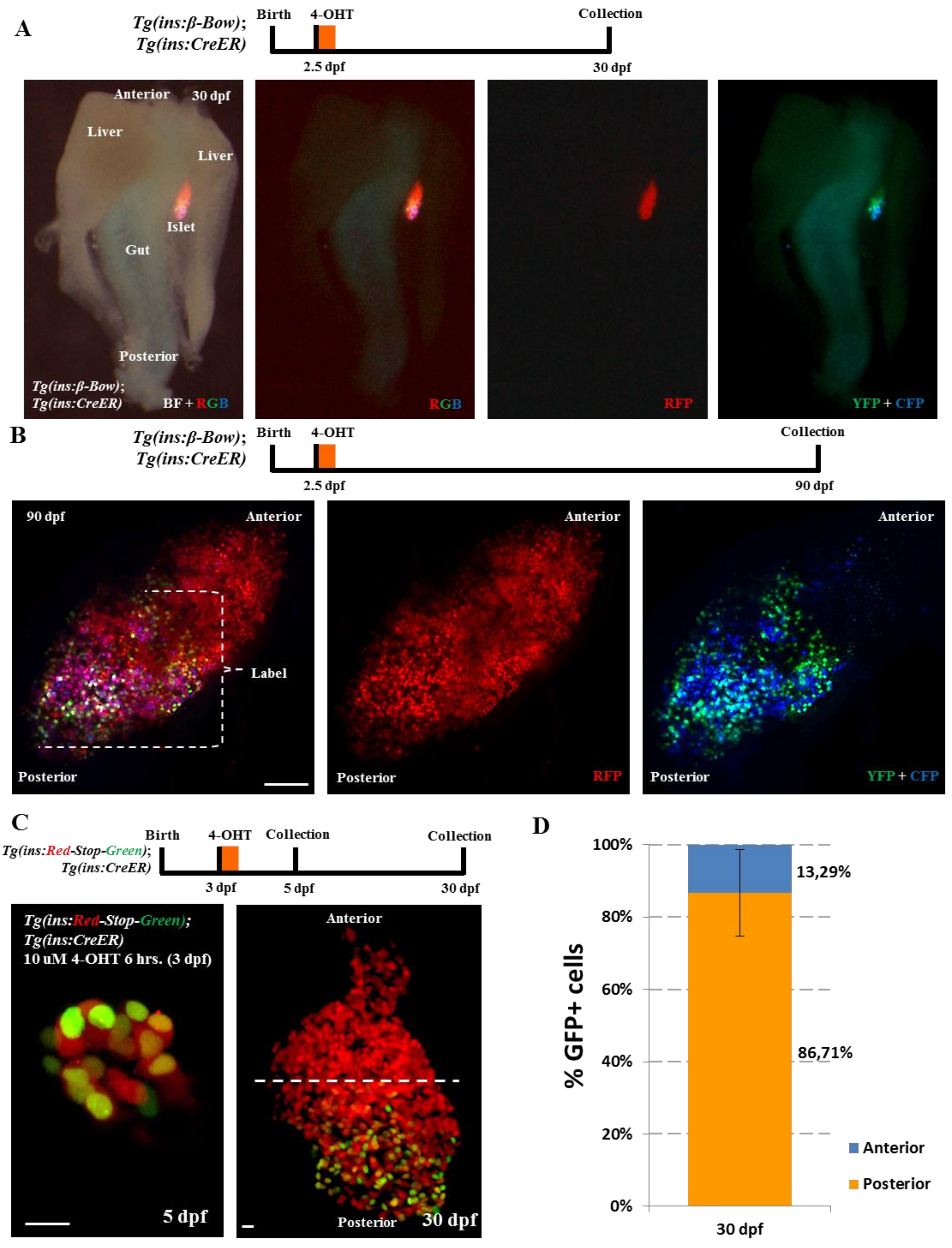

Supplementary Figure 11: Genetic-tracing of embryonic beta-cells.
(A) Whole-mount of the pancreas, liver and the intestine dissected from *beta-bow* animals at 30 dpf. Embryos were incubated with 4-OHT at 2.5 dpf in order to induce recombination and multicolor labeling of beta-cells. The bright-field (BF) overlaid with fluorescence images shows the localization and orientation of the zebrafish primary islet. The islet lies between the gut and one of the two lobes of the liver. The multicolor beta-cells undergoing recombination at 2.5 dpf (YFP+CFP-positive cells) exhibit predominant localization near the posterior regions of the islet at 30 dpf, whereas the anterior region is composed of cells exhibiting the default red-fluorescence of *beta-bow*. Anterior is to the top of the images.

(B) Maximum intensity projection of islets from 90 dpf (adult) *beta-bow* animals. Embryos were incubated with 4-OHT at 2.5 dpf. The beta-cells undergoing recombination at 2.5 dpf (YFP+CFP-positive cells) cluster near the posterior regions of the adult islet at 90 dpf, whereas the anterior region contains un-recombined cells, exhibiting the default red-fluorescence of *beta-bow*.

(C) Top – schematic for tracing of embryonic beta-cells during growth. *Tg(ins:mCherry-Stop-H2B-EGFP); Tg(ins:CreER^{T2})* animals were incubated with 10 uM 4-OHT at 3 dpf in order to label the embryonic beta-cells with nuclear GFP fluorescence. Samples were collected at 5 and 30 dpf.

Bottom - representative maximum intensity projection of primary islets from recombined *Tg(ins:mCherry-Stop-H2B-EGFP); Tg(ins:CreER^{T2})* animals at 5 and 30 dpf. Dashed line marks the center of the 30 dpf islet's anterior-posterior axis. At 5 dpf, 53 ± 12% of the beta-cells were GFP+ (n = 11 islets).

(D) Quantification showing the percentage of GFP+ beta-cells present within the anterior and posterior region of the 30 dpf islets. 86 ± 12 % of the GFP+ beta-cells were located in the posterior half of the islet, while 13 ± 12 % of the GFP+ beta-cells were located in the anterior half (n = 7 islets). Error bars = S.D. Scale bar in B, 100 µm; 10 µm in C.

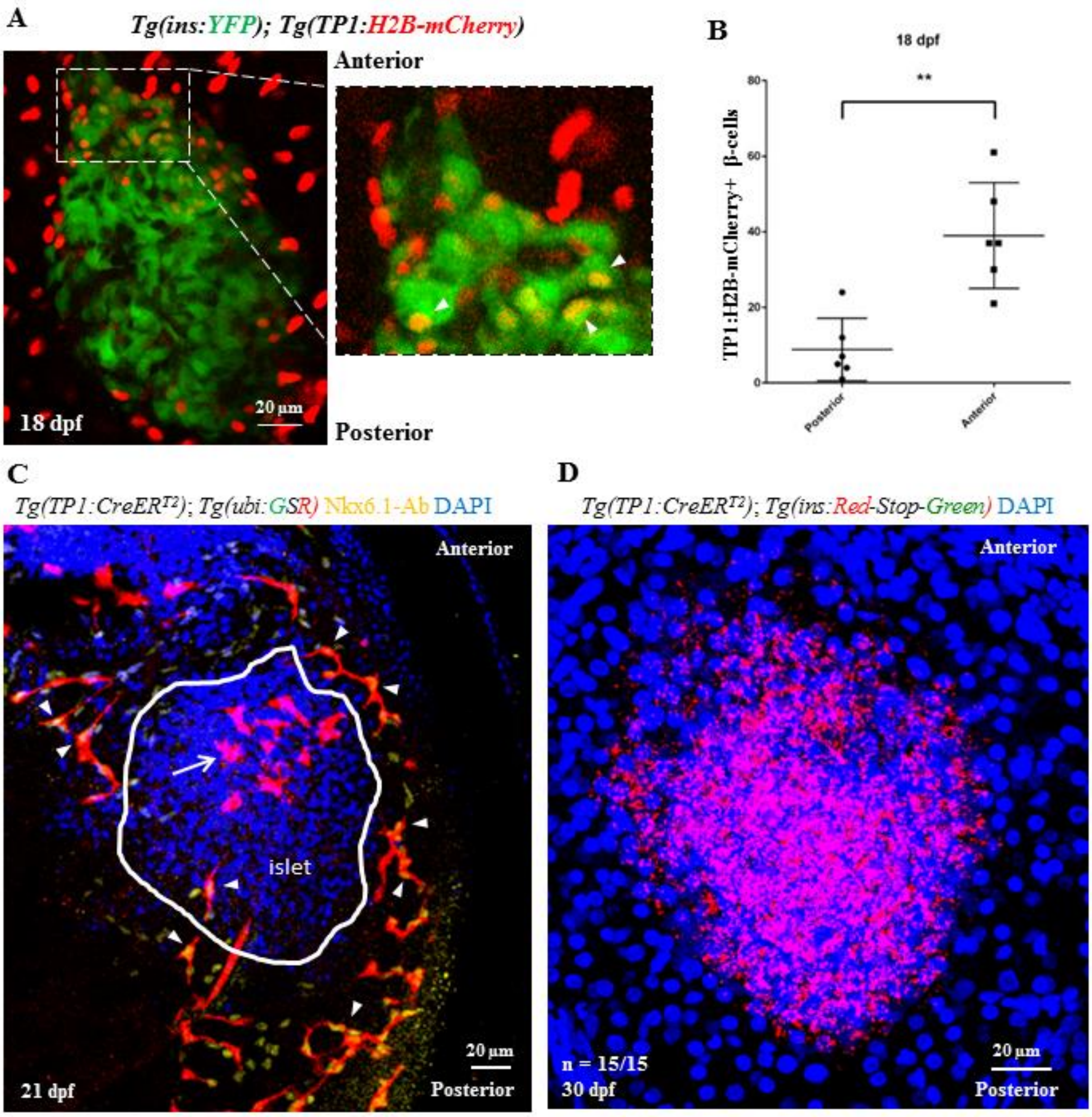

Supplementary Figure 12: Lineage tracing of Notch-responsive cells.
(A) Short-term lineage tracing of the Notch-responsive cells (NRCs) using histone-tagged mCherry. Maximum intensity projections of the primary islet from 18 dpf *Tg(TP1:H2B-mCherry);Tg(ins:YFP)* animals. *Tg(TP1:H2B*-mCherry)-expression labels the NRCs and *Tg(ins:*YFP*)* labels the beta-cells. The inset shows a higher magnification image of the anterior region of the islet. Due to the slow turnover of histone tagged proteins, *Tg(Tp1:*H2B-mCherry*)*$^{+}$ progenitors retain the fused fluorescent protein upon differentiating into beta-cells, while at the same time they activate the *insulin* promoter. Thus, NRCs differentiating into beta-cells are co-labeled with YFP and H2B-mCherry (arrowheads in the inset).
(B) Quantification of the short-term lineage tracing experiment. The plot shows the data points overlaid with a bar depicting Mean ± S.D. The anterior half of the islet harbors more YFP and H2B-mCherry-double positive cells as compared to the posterior half (t-test, ** $p \leq 0.01$).
(C) Uniform labeling of pancreatic ductal cells with *Tg(Tp1:CreER^{T2})*. Maximum intensity projection of the pancreata from 21 dpf *Tg(Tp1:CreER^{T2}); Tg(-3.5ubb:loxP-EGFP-loxP-mCherry)* animals incubated with 4-OHT at 4 dpf to induce recombination in the NRCs. Nkx6.1 immunofluorescence labels all ductal cells and DAPI labels cell nuclei. NRCs with successful recombination exhibit mCherry expression (red) and co-localize with the Nkx6.1^{+}

cells (arrowheads) on all sides of the primary islet without any anterior/posterior bias. Note the presence of mCherry-positive and Nkx6.1-negative cells (arrow) within the anterior half of the primary islet (outlined by a white line), which likely represent NRCs that differentiated into the endocrine lineages.
(D) Lack of leaky recombination in *Tg(Tp1:CreER*^{T2}*)* animals at 30 dpf. Maximum intensity projection of the primary islets of *Tg(Tp1:CreER*^{T2}*); Tg(ins:mCherry-Stop-H2B-EGFP)* animals incubated in vehicle at 4 dpf and stained with an anti-GFP antibody. Cells with leaky recombination would show green fluorescence. All samples (n = 15) lacked green fluorescence, suggesting absence of background recombination. Scale bars, 20 µm.

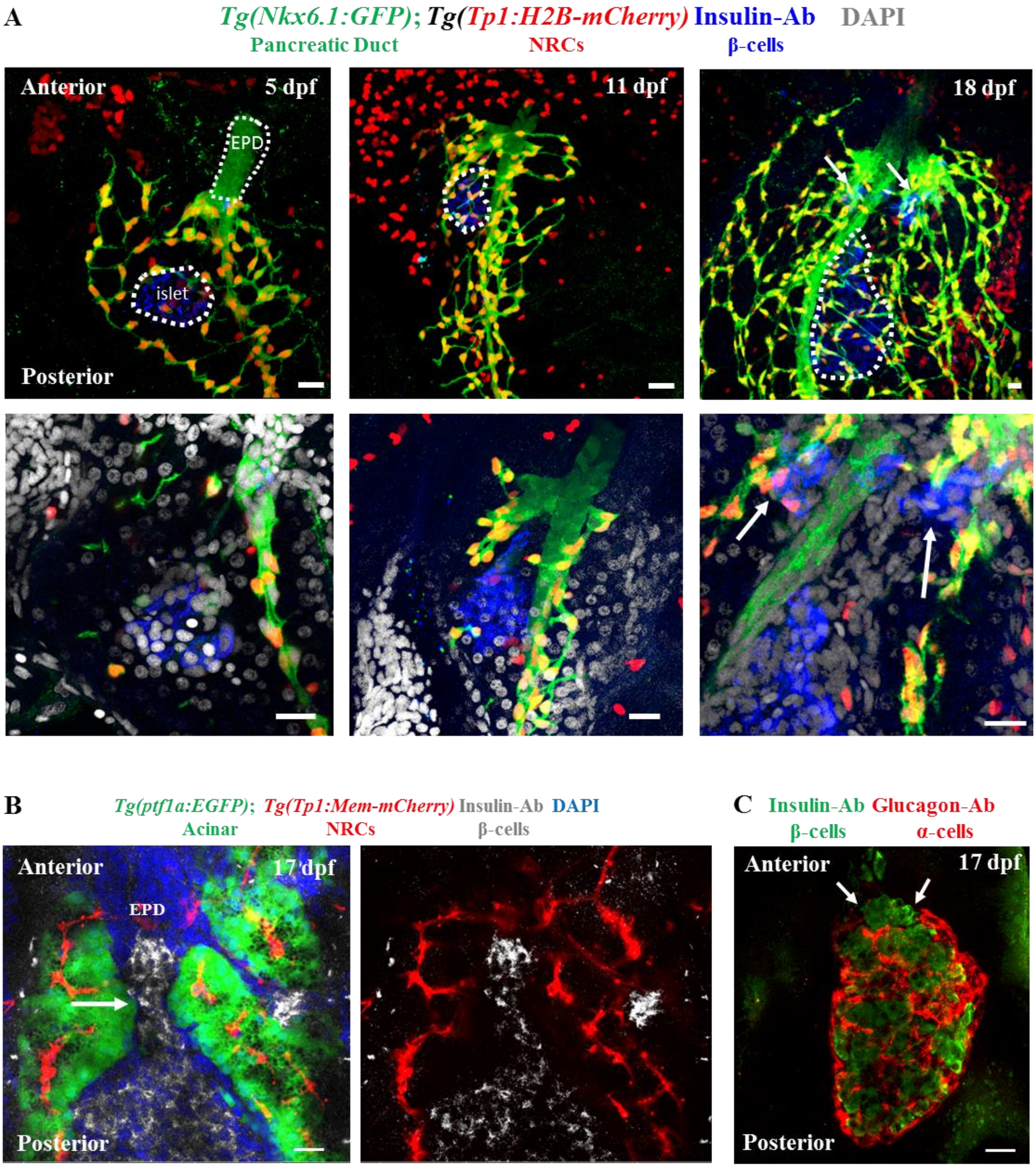

Supplementary Figure 13: Beta-cells that differentiate from post-embryonic progenitors coalesce with the anterior side of the principal islet.

(A) Time course analysis of beta-cells and beta-cell progenitors at 5, 11, and 18 dpf. The progenitor population consists of Notch-responsive cells (NRCs) within the ductal lineage. *Tg(Tp1:H2B-mCherry)* marks the NRCs (red), while the entire ductal linage exhibits expression of *Tg(Nkx6.1:*GFP*)* (green). Insulin immunofluorescence marks the beta-cells. The primary islet is outlined in the maximum intensity projections (top panels). The bottom panels represent single-plane images at higher magnification. Note the lack of NRCs within the extra-pancreatic duct (EPD, outlined at 5 dpf). Arrows at 18 dpf indicate two clusters of beta-cells in proximity to the anterior side of the primary islet.

(B) Maximum intensity projection of the pancreata of 17 dpf *Tg(ptf1a:EGFP)*; *Tg(Tp1:mem-mCherry)* animals stained for insulin and DAPI. Acinar cells were labeled by *Tg(ptf1a:*EGFP*)*-expression and NRCs were labeled by *Tg(Tp1:mem*-mCherry*)*-expression. A cluster of beta-cells is transitioning

between the acinar cells and appears to coalesce with the anterior side of the primary islet (arrow). **(C)** Maximum intensity projections of the pancreata of 17 dpf animals stained with anti-glucagon and anti-insulin antibodies to mark alpha- and beta-cells, respectively. Alpha-cells form the outer layer of the islet and surround the beta-cells, which are located inside the islet. The anterior side of the islet exhibits an opening within the alpha-cell layer (arrows), which is filled with the beta-cells incorporating into the anterior side of the primary islet. Note that the alpha-cell layer remains un-interrupted along the lateral and posterior sides of the islet. Scale bars, 20 μm.

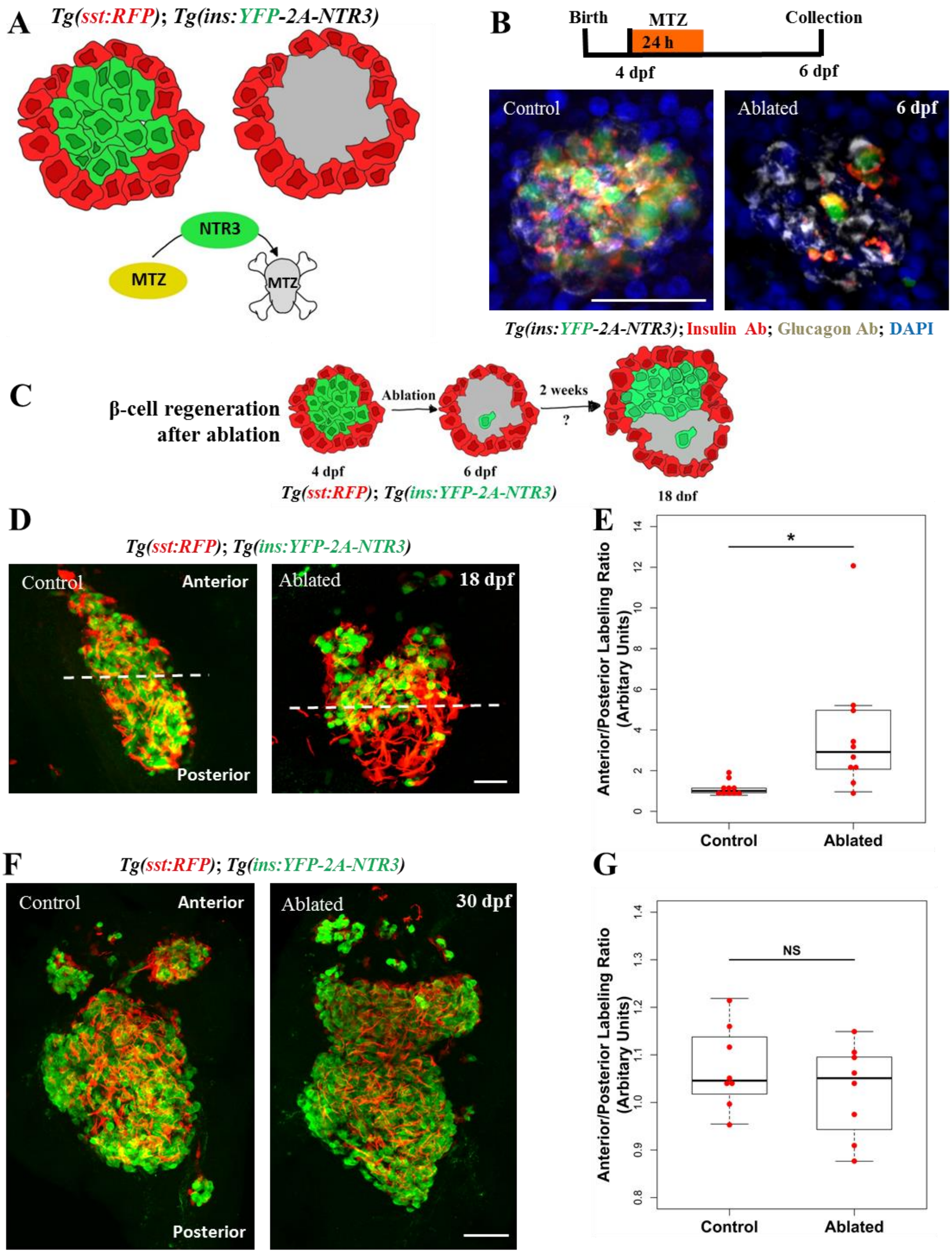

Supplementary Figure 14: Regeneration of beta-cells is asymmetric along the anterior-posterior axis of the primary islet.

(A) Cartoon depicting the experimental setup for conditional ablation of beta-cells. *Tg(ins:YFP-2A-NTR3)* drives beta-cell specific expression of a triple-mutant version of the Nitroreductase (NTR) enzyme, which converts the substrate metronidazole (Mtz) into a cytotoxic substance with higher efficiency compared to wild-type NTR. Mtz-treatment kills specifically the NTR-expressing beta-cells without harming their neighbors. *Tg(sst:*RFP*)*-expression in delta-cells is used to outline the perimeter of the islet (red).

(B) Top - schematic for testing the efficiency of beta-cell ablation upon Mtz-treatment. Animals were incubated in 10 mM Mtz (or vehicle) at 4 dpf for 24h and their pancreata were analyzed 48h later (6 dpf).
Bottom - maximum intensity projections of the pancreata of control and Mtz-treated (ablated) *Tg(ins:YFP-2A-NTR3)* animals stained with anti-insulin and anti-glucagon antibodies. DAPI labels cell nuclei. The MTZ-treated animals showed marked loss of beta-cells, while glucagon-expressing alpha-cells were spared from the ablation.
(C) Cartoon depicting the experimental setup for evaluating the pattern of beta-cell regeneration following the near-complete ablation of the embryonic beta-cells. Beta-cells (green) were ablated using *Tg(ins:YFP-NTR3)*. *Tg(sst:*RFP*)*-expression in delta-cells helps outline the islet's perimeter (red). Following ablation at 4 dpf, the pattern of regeneration is assessed at 18 dpf.
(D) Maximum intensity projections of islets oriented along the anterior-posterior (A/P) axis from control and beta-cell-ablated *Tg(ins:YFP-NTR3);Tg(sst:RFP)* animals at 18 dpf (14 days post-ablation). The control islets exhibited uniform beta-cell mass, while in the regenerating islets, a majority of beta-cells were confined to the anterior region. The filamentous appearance of RFP-fluorescence is a result of fixation artifacts that are commonly observed with RFP in this transgenic line.
(E) Quantification of the localization of YFP+ beta-cells within the anterior and posterior halves of the control and regenerating islets. The plot shows tukey style boxplot overlaid with the data points. The ratio of intensity of YFP-fluorescence in the anterior versus the posterior half is used as a proxy to estimate the abundance of beta-cells in each half. A ratio of one suggests similar beta-cell occupancy within the two halves, while a greater number indicates more cells in the anterior region. The un-ablated islets exhibited nearly equal beta-cell distribution in the two halves, whereas the regenerating islets showed more beta-cells in the anterior half (t-test, * $p \leq 0.05$).
(F) The control and ablated animals were allowed to recover for 26 days (30 dpf). Maximum intensity projections of control and Mtz-treated *Tg(ins:YFP-NTR3);Tg(sst:RFP)* animals. DAPI labels cell nuclei. The ablated islets exhibited uniform localization of beta-cells throughout the islet.
(G) Quantification of beta-cell abundance within the anterior and posterior halves of the primary islet for the controls and the ablated animals at 30 dpf. The plot shows tukey style boxplot overlaid with the data points. 8 islets were analyzed in each case. No significant differences (t-test, NS $p > 0.05$) were found between the two halves, suggesting uniform regeneration of beta-cells throughout the islet. Scale bars, 50 μm.

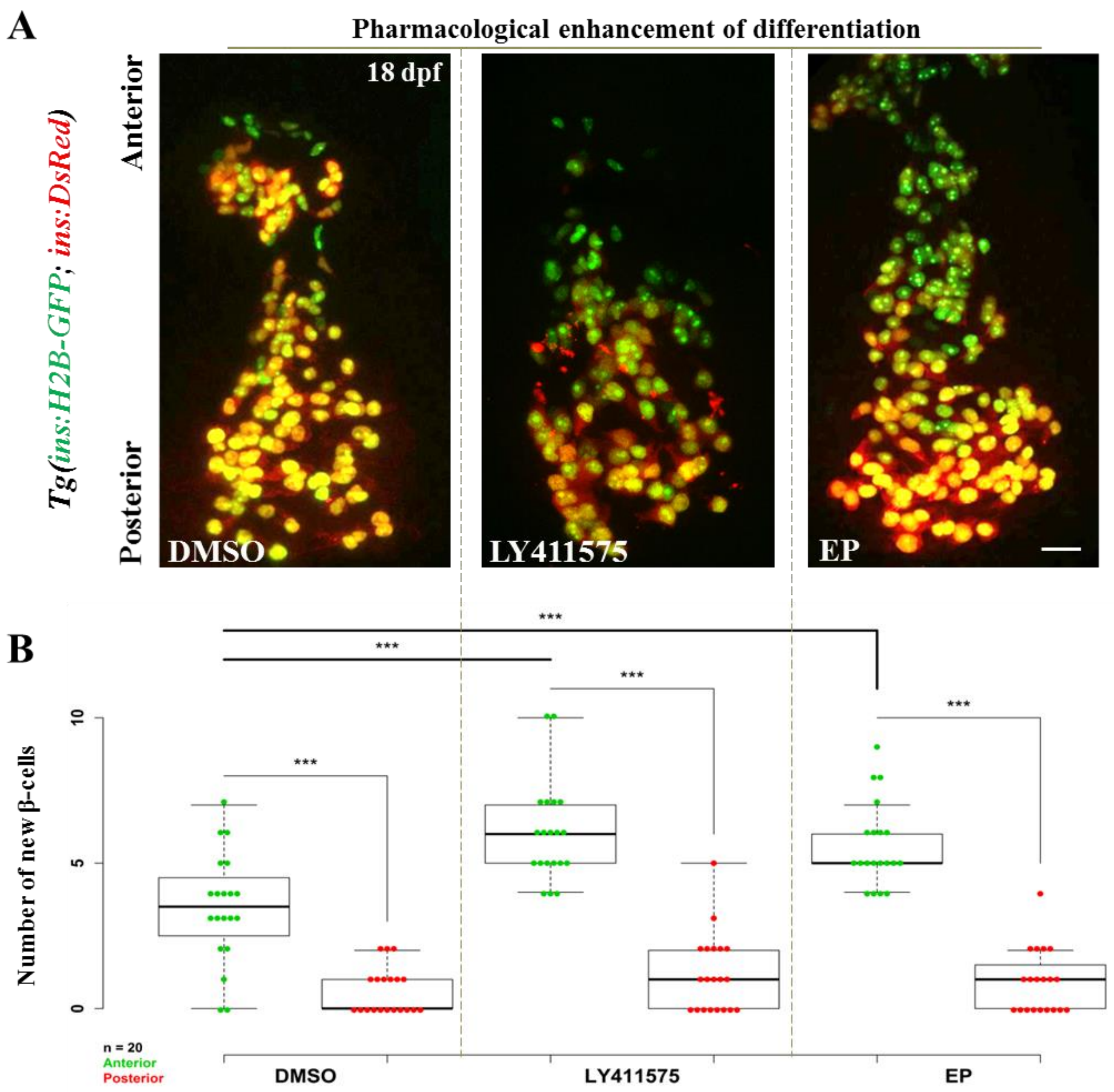

Supplementary Figure 15: Pharmacological inhibition of Notch and NF-kB signaling increases the number of recently-differentiated cells on the islet's anterior side.

(A) *Tg(ins:H2B-GFP);Tg(ins:DsRed)* animals were treated with DMSO (control), a γ-secretase inhibitor (LY-411575) or an NF-kB inhibitor (Ethyl Pyruvate (EP)), and the number of recently-differentiated beta-cells was quantified. The recently-differentiated beta-cells are H2B-GFP-positive but DsRed-negative. Maximum intensity projections of 18 dpf primary islets. The anterior side is to the top.

(B) Quantification of recently-differentiated beta-cells within the anterior and posterior halves of the primary islet (n = 20 islets) for controls and animals treated with LY-411575 or EP. The plot shows tukey style boxplot overlaid with the data points. In all three cases, the number of recently-differentiated beta-cells was higher within the anterior compared to the posterior half (two-tailed t-test, *** $p < 0.001$). Treatment with LY-411575 or EP significantly enhanced (two-tailed t-test, *** $p < 0.001$) the addition of beta-cells within the anterior halves of the islets, as compared to controls, but not within the posterior. Scale bars, 20 µm.

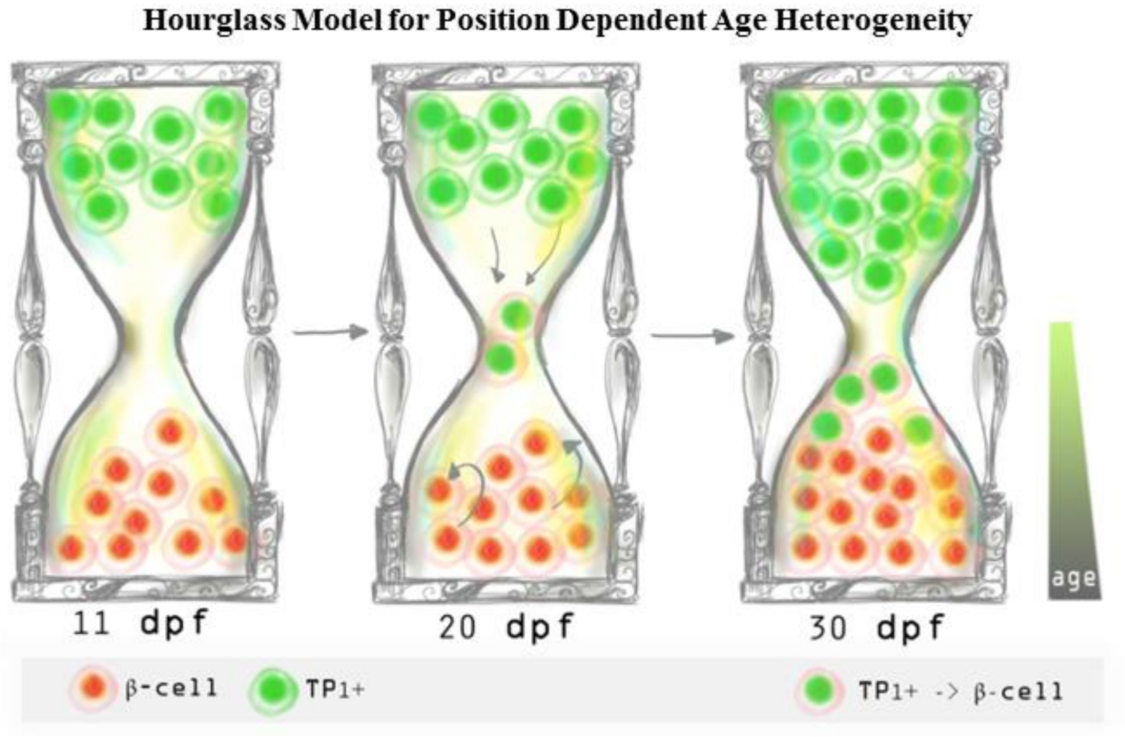

Supplementary Figure 16: An "hourglass" model for primary islet development. A model summarizing the asymmetric contribution of post-embryonic progenitor cells to the growth of the primary islet. The pre-existing beta-cell mass (red) is supplanted by addition of new beta-cells via differentiation of Notch-responsive cells on the anterior side of the islet. This differentiation leads to a gradient of age along the A/P axis of the primary islet. The cells at the bottom of the islet (corresponding to old sand in an hourglass) are derived from embryonic progenitors (and undergo proliferation to enhance cell mass); beta-cell differentiation from post-embryonic progenitors contributes to the top of the islet (anterior).

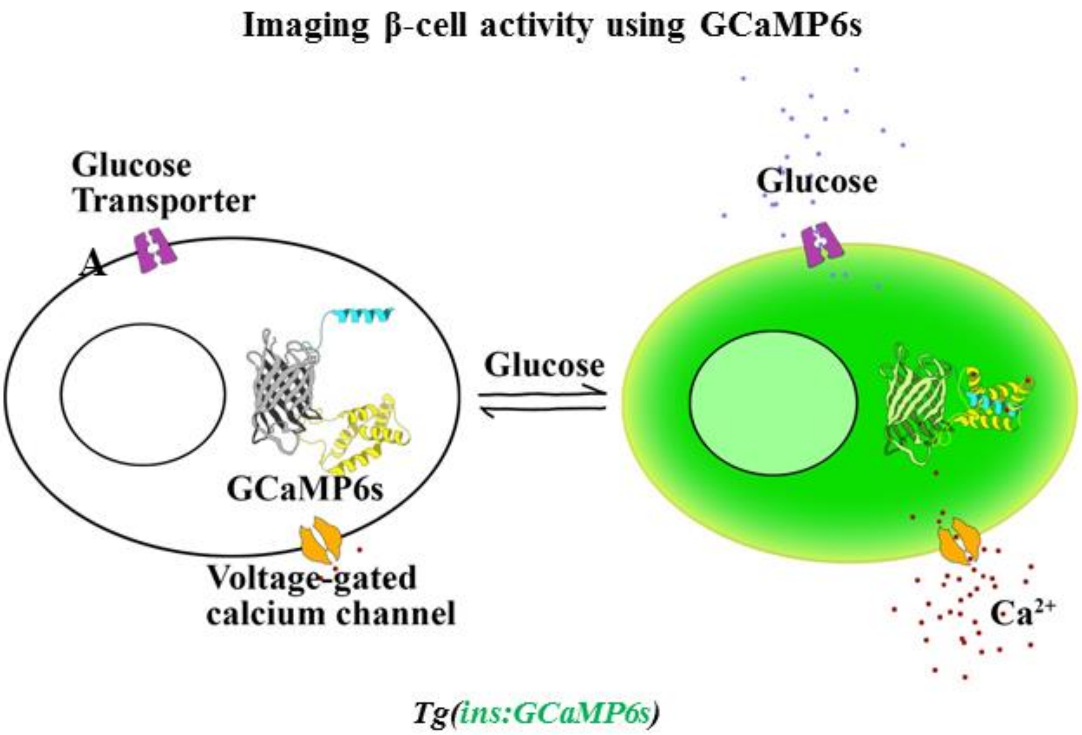

Supplementary Figure 17: Method for visualizing the glucose-stimulated calcium flux in beta-cells using a beta-cell-specific GCaMP6s.

Schematic representation for studying the glucose-induced calcium influx in beta-cells using the genetically-encoded calcium indicator GCaMP6s. Glucose-stimulation leads to an influx of Ca^{2+} in beta-cells. Intracellular Ca^{2+} binds GCaMP6s, leading to conformational change and an increase in the emission of green fluorescence. *Tg(ins:GCaMP6s)* drives GCaMP6s specifically in beta-cells under the control of the *insulin* promoter.

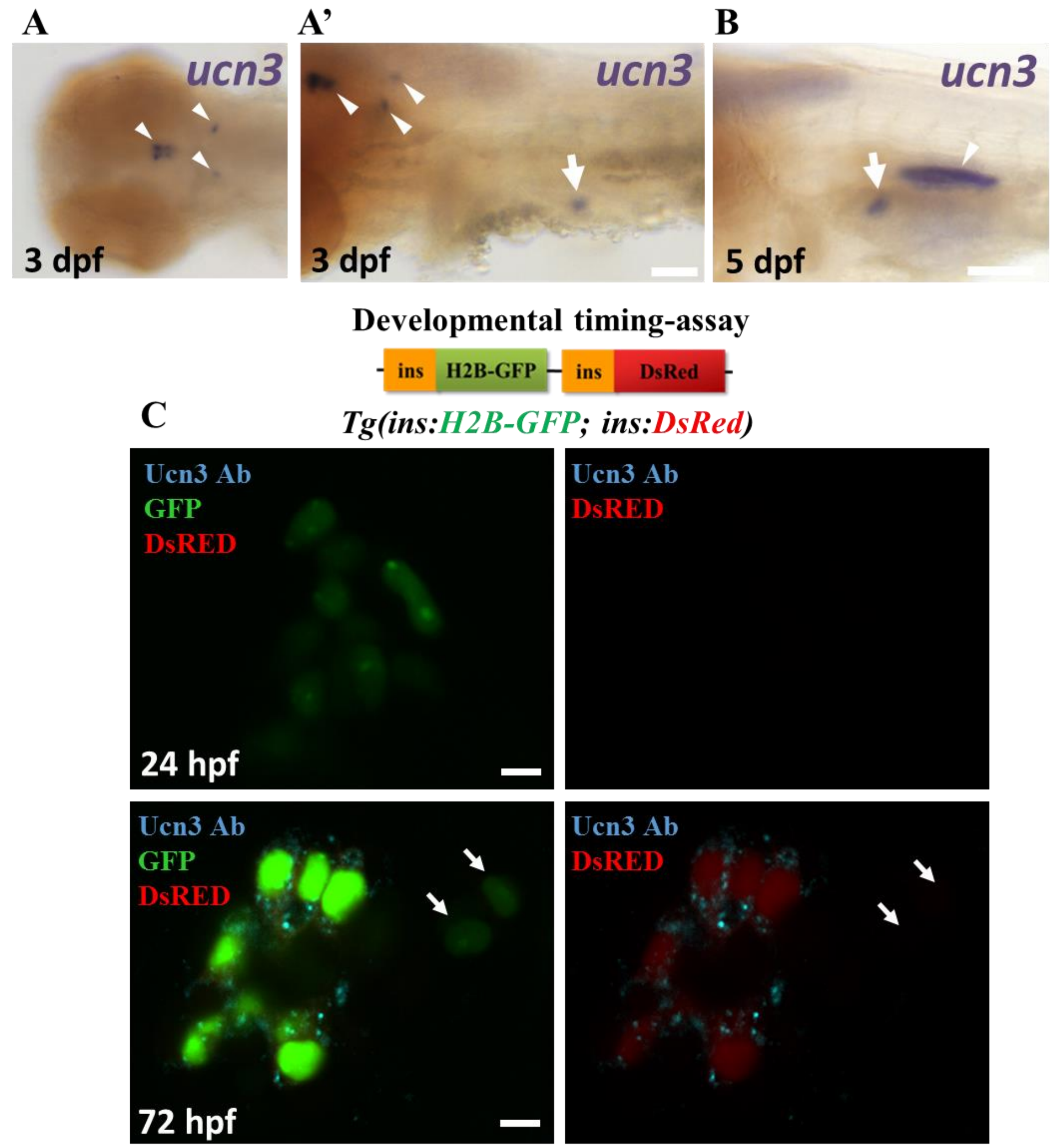

Supplementary Figure 18: *ucn3* expression in zebrafish embryos and larvae.
(A-B) Whole mount in-situ hybridization for *ucn3* (purple). **(A)** At 3 dpf, *ucn3* transcripts are expressed in characteristic groups of neurons in the brain (arrowheads in A and A'), consistent with a previous report[1]. In addition, *ucn3* is expressed in the islet (arrow in A'). **(B)** An arrow points to the expression of *ucn3* in the islet at 5 dpf. *Ucn3* might also show expression in the cells forming the swim-bladder (arrowhead in B).
(C) Primary islets from *Tg(ins:H2B-GFP; ins:DsRed)* embryos at 24 and 72 hpf. EGFP and DsRed are both expressed under the *insulin* promoter. The relatively faster maturation of GFP compared to DsRED allows to transiently mark the recently-differentiated beta-cells with only green fluorescence and to distinguish them from older beta-cells, which express both colors. At 24 hpf, the GFP-positive cells lack detectable Ucn3-immunofluorescence, whereas at 72 hpf, a majority of the GFP-positive cells are Ucn3-positive. The arrows point to two newborn beta-cells (H2B-GFP-positive/DsRed-negative cells) adjacent to the islet. These cells are Ucn3-negative. The images represent single confocal planes, which were acquired using the same confocal settings at both 24 and 72 hpf. Scale bars in A and B, 1 mm; 5 µm in C.

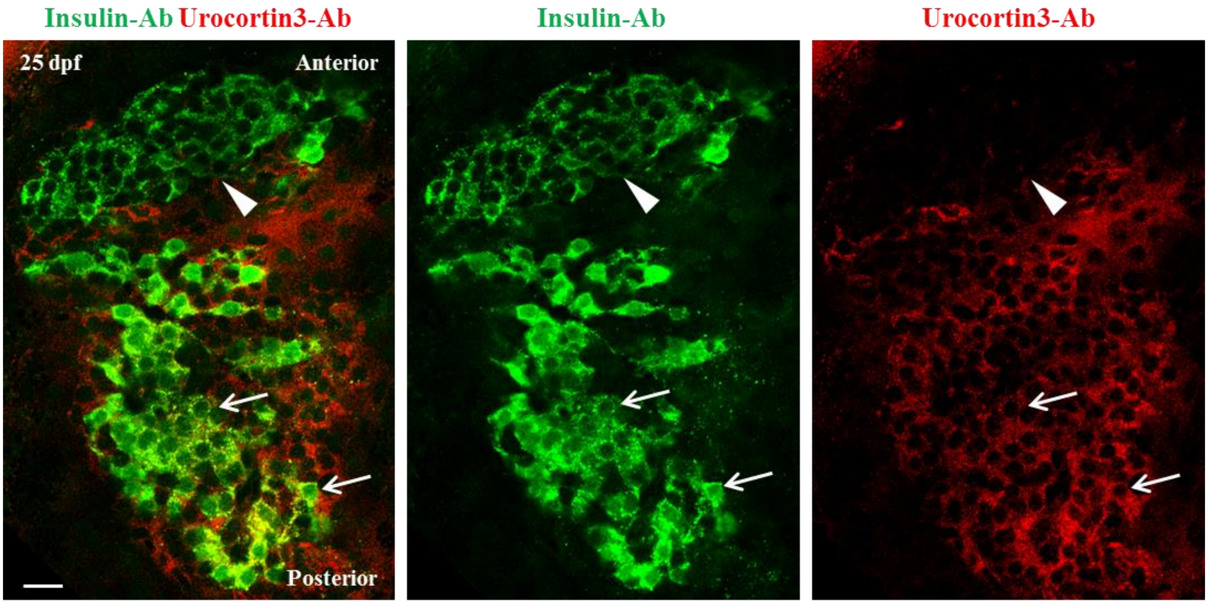

Supplementary Figure 19: A gradient of Urocortin3 expression along the A/P axis of primary islets at 25 dpf.

A single confocal plane of a primary islet from wild-type animals stained using antibodies against insulin and mouse-Urocortin3 (polyclonal) at 25 dpf. The islet is aligned along A/P axis (anterior is to the top). Beta-cells in the posterior region of the islet exhibit positivity for both insulin (green) and Urocortin3 immunofluorescence (red) (arrows). In contrast, the insulin-positive cells in the anterior region are Urocortin3-negative (arrowhead). Scale bars, 20 µm.

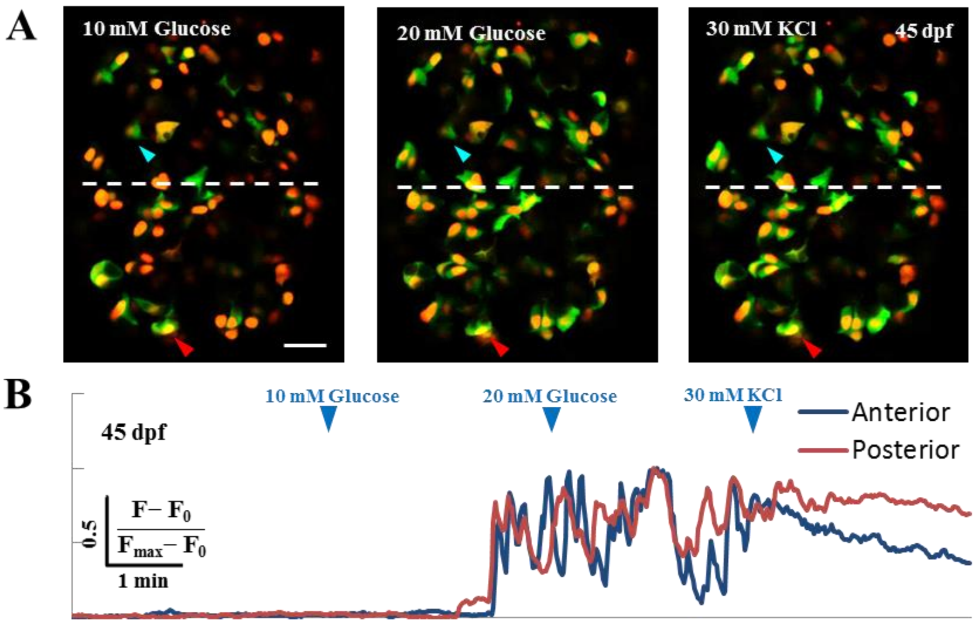

Supplementary Figure 20: Glucose-stimulated calcium fluxes in beta-cells at 45 dpf. **(A-B)** Glucose-responsiveness of larval beta-cells at 45 dpf. **(A)** Islets from *Tg(ins:GCaMP6s)*; *Tg(ins:mKO2-nls)* animals were mounted ex-vivo and live-imaged while providing increasing concentration of glucose from 5 mM (basal) to 10 and 20 mM, followed by addition of 30 mM KCl. The islets were divided into two halves along the anterior-posterior (A/P) axis, as indicated by the white dotted line (anterior is to the top). Representative cells within each half are marked with arrowheads (anterior – blue; posterior – red). **(B)** The fluorescence-trace for both cells is shown below in their respective colors. Both cells responded to the stimulation with glucose, as indicated by the oscillating GCaMP6s fluorescence-intensity. Scale bars, 20 µm.

Supplemental References:

1. Bräutigam, L., Hillmer, J. M., Söll, I. & Hauptmann, G. Localized expression of urocortin genes in the developing zebrafish brain. *J. Comp. Neurol.* **518,** 2978–95 (2010).
